# Association between *TCF7L2 *gene polymorphisms and susceptibility to Type 2 Diabetes Mellitus: a large Human Genome Epidemiology (HuGE) review and meta-analysis

**DOI:** 10.1186/1471-2350-10-15

**Published:** 2009-02-19

**Authors:** Yu Tong, Ying Lin, Yuan Zhang, Jiyun Yang, Yawei Zhang, Hengchuan Liu, Ben Zhang

**Affiliations:** 1 Open laboratory, West China Second University Hospital, Sichuan University, Chengdu 610041, PR China; 2 The Sichuan provincial key laboratory for human disease gene, Sichuan Academy of Medical Sciences and Sichuan Provincial People's Hospital, Chengdu 610072, PR China; 3 Department of Community Health, the Health Bureau of Wuhou District, Chengdu 610041, PR China; 4 Department of Epidemiology & Public Health, Yale University School of Medicine, 60 College Street, LEPH 440, New Haven, CT 06520, USA; 5 Department of Medical Laboratory, West China School of Public Health, Sichuan University, Chengdu 610041, PR China; 6 Department of Laboratory Medicine, Sichuan Academy of Medical Sciences and Sichuan Provincial People's Hospital, Chengdu 610072, PR China

## Abstract

**Background:**

Transcription factor 7-like 2 (*TCF7L2*) has been shown to be associated with type 2 diabetes mellitus (T2MD) in multiple ethnic groups in the past two years, but, contradictory results were reported for Chinese and Pima Indian populations. The authors then performed a large meta-analysis of 36 studies examining the association of type 2 diabetes mellitus (T2DM) with polymorphisms in the *TCF7L2 *gene in various ethnicities, containing rs7903146 C-to-T (IVS3C>T), rs7901695 T-to-C (IVS3T>C), a rs12255372 G-to-T (IVS4G>T), and rs11196205 G-to-C (IVS4G>C) polymorphisms and to evaluate the size of gene effect and the possible genetic mode of action.

**Methods:**

Literature-based searching was conducted to collect data and three methods, that is, fixed-effects, random-effects and Bayesian multivariate mete-analysis, were performed to pool the odds ratio (*OR*). Publication bias and study-between heterogeneity were also examined.

**Results:**

The studies included 35,843 cases of T2DM and 39,123 controls, using mainly primary data. For T2DM and IVS3C>T polymorphism, the Bayesian *OR *for TT homozygotes and TC heterozygotes versus CC homozygote was 1.968 (95% credible interval (*CrI*): 1.790, 2.157), 1.406 (95% *CrI*: 1.341, 1.476), respectively, and the population attributable risk (PAR) for the TT/TC genotypes of this variant is 16.9% for overall. For T2DM and IVS4G>T polymorphism, TT homozygotes and TG heterozygotes versus GG homozygote was 1.885 (95%*CrI*: 1.698, 2.088), 1.360 (95% *CrI*: 1.291, 1.433), respectively. Four *OR*s among these two polymorphisms all yielded significant between-study heterogeneity (P < 0.05) and the main source of heterogeneity was ethnic differences. Data also showed significant associations between T2DM and the other two polymorphisms, but with low heterogeneity (*P *> 0.10). Pooled *OR*s fit a codominant, multiplicative genetic model for all the four polymorphisms of *TCF7L2 *gene, and this model was also confirmed in different ethnic populations when stratification of IVS3C>T and IVS4G>T polymorphisms except for Africans, where a dominant, additive genetic mode is suggested for IVS3C>T polymorphism.

**Conclusion:**

This meta-analysis demonstrates that four variants of *TCF7L2 *gene are all associated with T2DM, and indicates a multiplicative genetic model for all the four polymorphisms, as well as suggests the *TCF7L2 *gene involved in near 1/5 of all T2MD. Potential gene-gene and gene-environmental interactions by which common variants in the *TCF7L2 *gene influence the risk of T2MD need further exploration.

## Background

Type 2 diabetes mellitus (T2DM) is characterized by hyperglycemia that can occur through mechanisms such as impaired insulin secretion, insulin resistance in peripheral tissues and increased glucose output by liver [1]. Most individuals with T2DM suffer serious complications of chronic hyperglycemia, involved in nephropathy, neuropathy, retinopathy, and accelerated development of cardiovascular disease [2]. The prevalence of T2DM has increased sharply during the past two decades in the world and is close to 6% [3]. But estimations suggest that this trend will continue to rise over the next decade owing to increasing age of population and surge of obesity [4]. Accounting for 90~95% of populations with diabetes, T2DM is projected to 300 million worldwide by year 2025 [2,4], and its incidence exhibits significant geographic and racial difference, ranging from more than 0.3 to 17.9% in Africa [5-11], 1.2 to 14.6% in Asia [12-19], about 2.5% in Australia [20], 0.7 to 11.6% in Europe [21-32], 4.6 to 40% in the Middle East [33-36], 6.69 to 28.2% in North America [3,37-39], and 2.01 to 17.4% in South America [40-43]. Recent years, new diagnosis of T2DM subjects in developing nations with a mass of populations such as China and India has increased rapidly [19,44,45], and it has also found remarkable augmented in rural that being with lower prevalence before [14,17,19,45]. Incidence rates are diverse in females and males in Europe, North America, Africa, Latin America and East Asia, and there are scarcely reports with accordant result.

Transcription factor 7-like 2 (*TCF7L2*) gene spans a 215,863 bases region on chromosome 10q25.3 [46] (114700201–114916063, NCBI build 36.2), and its product is a high-mobility box-containing transcription factor that has a role in activating many genes downstream of the Wnt signaling pathway and in T2DM [47-50]. The mechanisms through which TCF7L2 affects the susceptibility to T2DM remain to be elucidated. The bipartite transcription factor, cat/*TCF7L2*, activates many genes downstream of the Wnt signaling cascade [51]. One of the genes transcriptionally regulated by cat/*TCF7L2 *is proglucagon, which encodes the insulinotropic hormone glucagon-like peptide 1 (*GLP-1*) [49,50]. Because *GLP1*, in concert with insulin, plays a critical role in blood glucose homeostasis, it has been postulated that *TCF7L2 *gene variants may affect the susceptibility to type2 diabetes by indirectly altering *GLP-1 *levels [50].

The human *TCF7L2 *gene consists of 14 exons and 13 introns (NCBI build 36.2). However, a previous study [52] has shown that *TCF7L2 *has 17 exons, of which five are alternative. There are at least four well-studied polymorphic markers that have brought the most attention, that is, (1) a C-to-T (genomic position: 114748339) substitution at snp rs7903146 of the intron 3 (IVS3C>T) [2,47,53,54]; (2) a T-to-C (genomic position: 114744078) substitution at snp rs7901695 of the intron 3(IVS3T>C) [2,53,55]; (3) a G-to-T (genomic position: 14798892) substitution at snp rs12255372 of the intron 4 (IVS4G>T) [2,56]; and (4) a G-to-C (genomic position: 114797037)substitution at snp rs11196205 of the intron 4 (IVS4G>C) [57,58]. Most published epidemiologic studies have emphasized on the IVS3C>T and IVS4G>T polymorphisms [2,47,59-65]. There are plenty of evidence on strong linkage disequilibrium between these four polymorphisms [2,47,57,58]. The T allele of the IVS3C>T, C allele of the IVS3T>C, and T allele of IVS4G>T, as well as C allele of IVS4G>C polymorphisms have higher frequency in Caucasian [59,66], North European [2,47], African [67,68], and Indian [69,70] populations than those in East Asian [57,61,62,71] populations.

The polygenic basis of T2DM has been intensely investigated by large consortia worldwide, and consensus on a range of valid susceptibility genes has emerged. Such genes, for example *PPARG*, *KCNJ11 *and *CPN10*, appear to contain common variation that confers only 10–20% additional risk of T2DM, and only studies with large numbers of case and controls have had sufficient statistical power to examine their role robustly [72]. Recent years, some novel loci in genes such as *TCF7L2*, *SLC30A8*, *HHEX*, *ETX2*, *CDKN2A*/*CDKN2B*, *IGF2BP2*, *CDKAL1*, *FTO*, *PPARG*, and *KCNJ11 *have been found with susceptibility to T2DM [2,73-76], and these has promoted the development of genetics on T2DM. From 2006 to 2008, study on *TCF7L2 *gene polymorphisms and its association with T2DM has become the focus in diabetes mellitus, therefore, some meaningful results have reported one by one. In March, 2006, Grant and colleagues [2] first proposed an association with *TCF7L2 *gene variants with T2DM. The study [2] consisted of three samples from Iceland, Denmark and USA with strikingly similar results. Since then, this potential association has been examined in over 50 samples from around the world. Given the amount of accumulated data, it is important and urgent to perform a quantitative synthesis of these evidences. We therefore undertake a large meta-analysis of studies of the association between *TCF7L2 *gene polymorphisms and T2DM. To exclude heterogeneity of these studies, we stratified the data by Hardy-Weinberg equilibrium (HWE), publishing time, ethnicity, body mass index (BMI), diagnosis and age.

Although one meta-analysis [77] and several narrative reviews about *TCF7L2 *gene and T2DM had been reported [78-80], Succedent studies have now made more data available. Compared with the only one meta-analysis written by Cauchi and his colleagues [77] and published online in May, 2007, 28 more studies from 14 new papers [53,55,57,63,68,69,71,73-76,81-83]were available to us. And that, this meta-analysis of the association between *TCF7L2 *gene and T2DM, only summarized IVS3C>T polymorphism by just analyze allele frequency [77], the authors did not generalize the association between other important variations such as IVS4G>T, IVS3T>C and IVS4G>C polymorphisms and T2DM. Further, the only meta-analysis was based on published data, namely, without stratification, and, was thus limited in the interpretations they allowed. However, using the original data, we are able to explore more deeply, searching for sources of heterogeneity. Thus, we carried out a large meta-analysis to conduct a broad comprehensive and quantitative assessment on the association between variants of *TCF7L2 *gene and T2DM, and then, to perform a detailed examination of sources of heterogeneity. We sought to estimate magnitude of the genetic association as well as the genetic mode of action, and to determine the extent of heterogeneity in the strength of associations between different studies.

## Methods

### Search strategy and inclusion criteria

We conducted a comprehensive search of the US National Library of Medicine's PubMed, OMIM, ISI Web of Science, and Embase databases for all genetic association studies on at least one of the above four polymorphisms in the *TCF7L2 *gene and T2DM published from March, 2006, when the association between *TCF7L2 *gene and T2DM was first reported [2], through March, 2008. The search strategy was based on combination of the key words "transcription factor 7-like 2" or "*TCF7L2*" or "*TCF7L2 *gene polymorphism", and "diabetes" or "diabetes 2" or "type 2 diabetes" or "type 2 diabetes mellitus" or "T2D" or "T2DM". The references of all computer-identified publications were searched for additional studies, and the PubMed option "Related Articles" was used to search for potentially relevant papers. Reference lists in retrieved articles were also screened.

Searching was performed in duplicate by two independent reviewers (YZ and JYY). Without any language restriction (we will translate articles published in other languages into English by professional interpreters), we only selected published manuscripts (including their online supporting materials and the original data kindly sent by the authors) if they met the following criteria: 1) the publication was a population-based association study (family-based study design with linkage considerations was excluded), regardless of sample size, and 2) there were enough results for extraction of data, that is, the number of subjects with each allele and genotype in the T2DM and control groups. Where eligible papers had insufficient information, we contacted authors by e-mail or mail for additional information. In the case of sequential or multiple publications of analyses of the same data or overlapping data sets, the publication that reported data from the largest or most recent study was included.

### Data extraction

Data were extracted independently and in duplicate by two investigators (YZ and JJY) who used the recommended guidelines for reporting on meta-analyses of observational studies [84]. The following data were extracted from the eligible studies: authors, journal and year of publication, country of origin, selection and characteristics of cases and controls, demographic data, ethnicity of the study population (Caucasian, North European, East Asian, African, Middle Eastern, American, or Pacific Asian), numbers of eligible and genotyped cases and controls, and genotype distributions in cases and controls and available subgroups thereof. Furthermore, we examined whether matching had been used; whether there was specific mention of blinding of the genotyping personnel to the clinical status of subjects; whether the genotyping method used had been validated; and whether genotype frequencies in control groups were in HWE. Any disagreement was adjudicated by a third author (YWZ).

### Statistical Analysis

We used the odds ratio as the metric of choice and it was estimated for each study. In order to explore the possible association between *TCF7L2 *gene polymorphisms and T2MD and to avoid excessive comparisons, we calculated the odds ratio by two methods, namely, allele comparison (i.e., T allele vs. C allele in IVS3C>T polymorphism), and genotype comparison, that is, comparing the risk variant homozygotes and heterozygotes with wild homozygotes, respectively (i.e., TT vs. CC (*OR*_1_) and TC vs. CC (*OR*_2_) in IVS3C>T polymorphism; TT vs. GG (*OR*_3_) and TG vs. GG (*OR*_4_) in IVS4G>T polymorphism; CC vs. TT (*OR*_5_) and CT vs. TT (*OR*_6_) in IVS3T>C polymorphism; CC vs. TT (*OR*_7_) and CT vs. TT (*OR*_8_) in IVS4G>C polymorphism). We estimated and characterized the summary prevalence of risk allele by using only the data on controls. When we analyze genotype data for meta-analysis, cells with zero count had 0.5 been added. In addition, we calculated the population attributable risk (PAR) of risk allele according to method of Chang et al [71].

We first compared alleles for cases and controls in order to detect overall differences and genetic association. Allele frequencies were computed for studies reporting only genotypic data. Pooled odds ratio were computed three times, by the fixed-effects method of Mantel and Haenszel [85], by the random-effects method of DerSimonian and Laird [86], and by the Bayesian random-effects method of Warn et al [87]. Unless stated otherwise, random-effects estimates (DerSimonian and Laird) are reported here.

Our primary analysis for *TCF7L2 *gene polymorphisms and T2DM is based on genotype comparisons in order that magnitude of the genetic association and the genetic mode of action can be identified exactly. Once an overall gene effect was confirmed by the allele comparison, the genotype effects and genetic model were estimated by using the genetic model-free approach suggested by Minelli et al [88], where no assumptions about genetic models were required. The *OR*_1 _to *OR*_8 _were calculated by using multivariate meta-analysis with Bayesian method [88]. The logarithm (log) odds ratios were modeled on the base of both between and within study variations. Four separate stochastic lambda values (reflecting the genetic model), consisting of the ratios of log*OR*_2 _versus log*OR*_1 _for λ_1_, log*OR*_4 _versus log*OR*_3 _for λ_2_, log*OR*_6 _versus log*OR*_5 _for λ_3_, and log*OR*_8 _versus log*OR*_7 _for λ_4_, were calculated [88]. The parameter λ capture information about the genetic mode of action, briefly, the model is a recessive model if λ = 0, a codominant model if λ = 0.5, a dominant model if λ = 1, and homozygous or heterosis model if λ < 0 or λ > 1. Pooled odds ratio were also computed by the fixed-effects method of Mantel and Haenszel [85] and by the random-effects method of DerSimonian and Laird [86]. Unless stated otherwise, Bayesian estimates are reported here.

We tested for deviations from Hard-Weinberg equilibrium (HWE) in both case and control populations by using the exact method reported by Emigh et al [89] whether the author provide the *P *values of HWE or not. For above allele-based and genotype-base analyses, we took two methods to handle Hard-Weinberg disequilibrium. Firstly, we performed sensitivity analyses by including by including and excluding studies in Hard-Weinberg disequilibrium (case and control group were all considered). Secondly, we included all studies regardless of HWE and instead adjusted for the degree of disequilibrium by using the inbreeding coefficient (*F*) suggested by Trikalinos et al [90]. Briefly, the *F *was estimated for each study by using data in the control group. Predicted genotype frequencies were estimated [91] and used instead of the observed frequencies in the summary analysis of magnitude and genetic model.

In sensitivity analysis, we estimated between-study heterogeneity across all eligible comparisons using Cochran's *Q *statistic [92]. Heterogeneity was considered significant at *P *value less than 0.10 [92]. We also report *I*^2 ^statistic, which describes the percentage of variability in point estimates due to sample heterogeneity rather than sampling error [93,94], and can quantify heterogeneity irrespective of the number of studies [94,95]. As a guide, *I*^2 ^values less than 25 percent may be considered "low", values of 31 percent to 56 percent have been defined as indicating "low" to "moderate" heterogeneity, whereas values greater than 56 percent are considered indicative of "notable" heterogeneity, and "large" heterogeneity is stipulated for *I*^2 ^values of larger than or equal to 75 percent [94,95]. The examination of heterogeneity was performed separately for two odds ratios (*OR*_1 _and *OR*_2 _for IVS3C>T polymorphism, *OR*_3 _and *OR*_4 _for IVS4G>T polymorphism, *OR*_6 _and *OR*_7 _for IVS3T>C polymorphism, *OR*_7 _and *OR*_8 _for IVS4G>C polymorphism) in genotype-based analysis. If there was heterogeneity on at least one of these two odds ratios, the source of the heterogeneity was explored by fitting a covariable (such as age, gender, ethnicity, BMI) in a meta-regression model [96-98]. Possible subgroup analyses were involved in ethnicity (Caucasians, North Europeans, East Asians, Indians and other racial groups), age (< 65 years vs. ≥ 65 years), diagnosis (normal diagnosis vs. no diagnosis or false diagnosis), publishing time (2006 vs. 2007 and 2008) and BMI (normal (< 30.0 kg/m^2^) vs. abnormal (≥ 30.0 kg/m^2^) according to the meta-regression analysis. Logistic regression analysis was used to compare odds ratios among these possible groups. In addition, genetic association studies may be especially susceptibility to the selective publication of positive findings on gene association [99]. In order to investigate the publication bias among studies, funnel plots [100] and cumulative meta-analysis [101] were used.

All analyses were conducted in Stata software, version 9.0 [102], using *meta*, *metan*, *metabias*, *metacum*, and *metareg *commands, except for the Bayesian method of genotype-based analysis. We fit the Bayesian models by using Markov chain Monte Carlo (MCMC) methods with Bayesian framework and perform our inferences using WinBUGS 1.4.3 [103], taking advantage of its flexibility as well as its ability to incorporate full uncertainty in all unknown parameters. Bayesian analyses yield credible intervals rather than confidence intervals; a 95% credible interval (*CrI*) describes a range in which the probability that the unknown quantity lies within this interval, after seeing the data, is 95%. A 'burn-in' of 10,000 iterations is carried out for models, followed by 50,000 iterations for parameter estimates. A *P *value less than 0.05 was considered statistically significant, except for tests of heterogeneity, where a level of 0.10 was used.

## Results

### Eligible studies

Totally eighty-nine papers were identified across Medline and Embase on the base of our search strategies. Two of the studies [57,71]did not have genotypic data, but the authors friendly sent the supplementary information to us. Parra et al [68], reported four samples, that is, two Mexican American populations, one Spanish and one Nigerian population. With no controls of last three populations, we combined the two Mexican samples and then, discarded the Spanish and Nigerian samples in meta-analysis of polymorphism of IVS3C>T and IVS4G>T. Therefore, 25 eligible papers [2,53,55-71,74,75,77,81-83], including 36 studies (multiple studies performed in one paper in particular: 3 studies in paper of Grant et al [2], Horikoshi et al [62], Humphries et al [67], and Miyake et al [82]; 2 studies in paper of Scott et al [74], Sladek et al [75], and Cauchi et al [77]), examining the association between *TCF7L2 *gene polymorphisms and T2DM were identified by the inclusion criteria and all of them were written in English (Table 1, Additional file 1). The eligible studies for analysis involved in a total of 35,843 cases with T2DM and 39,123 controls. 35 studies from 24 papers [2,53,55,57-71,74,75,77,81-83] contained data for the IVS3C>T polymorphism (Table 2, Additional file 1), 10 studies from 8 papers contained data for the IVS3T>C polymorphism (Table 3, Additional file 1), 21 papers involved in 29 studies contained data for the IVS4G>T polymorphism (Table 4, Additional file 1), and 9 papers including 15 samples contained data for IVS4G>C polymorphism (Table 5, Additional file 1).

There was considerable diversity of ethnic groups. Eligibility criteria for T2DM patients were shown in Table 1 (Additional file 1). Controls were mainly healthy populations who were described as nondiabetic and/or, Normoglycemic, and/or normal glucose tolerance (NGT), although varying details were presented regarding the extent of testing that had been done to excluded controls with impaired glucose tolerance and diabetes (table 1, Additional file 1). Remaining 3 investigations did not report the details of controls [60,66,67]. 1 study matched for geographic region [53]; 1 study matched for age, race, and BMI [56]; 5 studies matched for race [55,67,70,83]; and one study 1 study matched for age, sex, and geographic region [58,65]. The other investigations did not provide for the details of the matching information. Detailed characteristics of each investigation, along with *P *values for testing HWE, are listed in the Table 2–5 (Addional file 1) for IVS3C>T polymorphism, IVS3T>C polymorphism, IVS4G>T polymorphism and IVS4G>C polymorphism, respectively.

### IVS3C>T polymorphism

The eligible studies for analysis of IVS3C>T polymorphism included a total of 33,135 cases with T2DM and 36,316 controls (Table 2, Additional file 1). Allele and genotype data were available for all the cases and controls in the eligible studies. There was no evidence of Hard-Weinberg disequilibrium in both cases and controls.

#### Bias diagnostics

**Figure 1:**
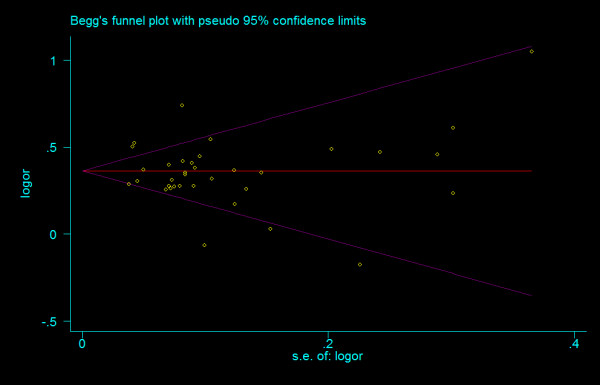
**Funnel plots of rs7903146 polymorphism studies for T allele vs. C allele (corrected *z *= 0.82, corrected *P *= 0.410)**.

**Figure 2:**
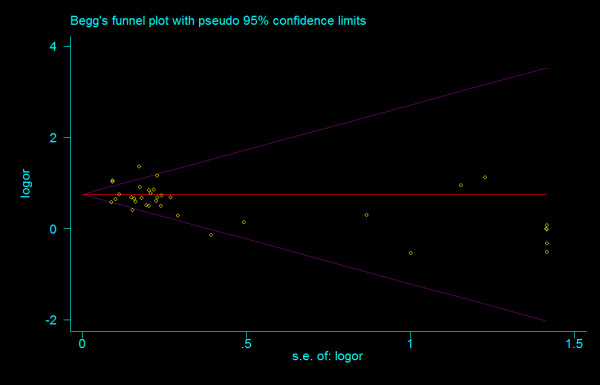
**Funnel plots of rs7903146 polymorphism studies for T homozygotes vs. C homozygotes (corrected *z *= 0.02, corrected *P *= 0.987)**.

**Figure 3:**
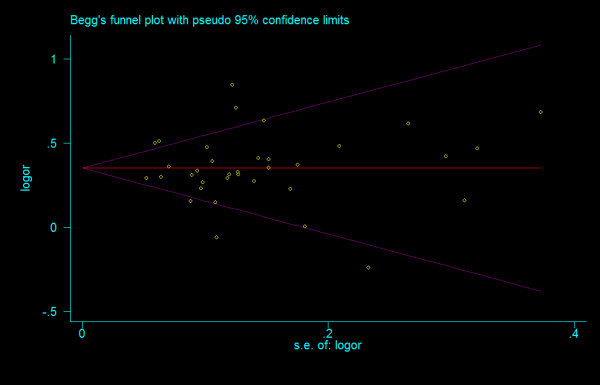
**Funnel plots of rs7903146 polymorphism studies for heterozygotes vs. C homozygotes (corrected *z *= 0.80, corrected *P *= 0.426)**.

**Figure 4:**
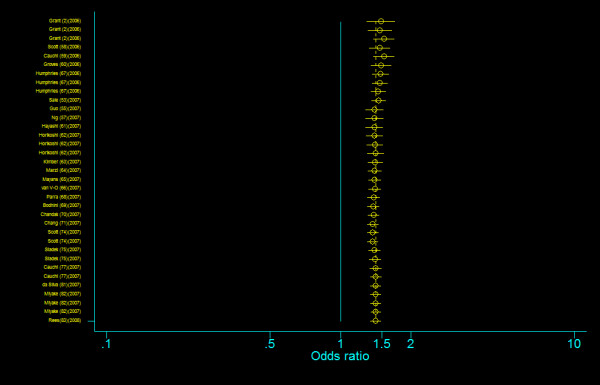
**Cumulative meta-analysis of the odds ratios of association between rs7903146 polymorphism of *TCF7L2 *gene and T2DM for T allele vs. C allele**.

**Figure 5:**
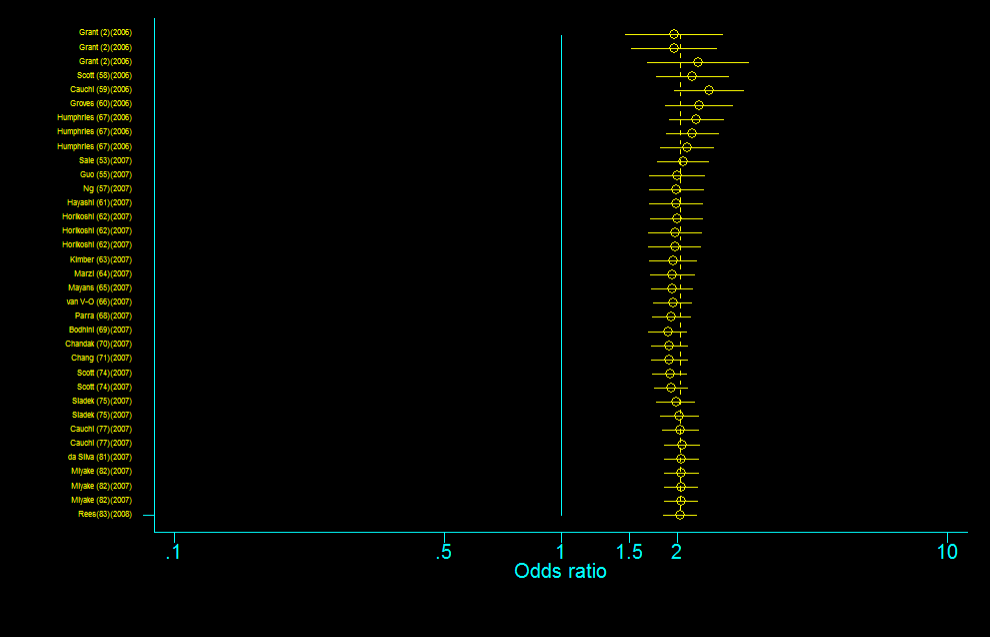
**Cumulative meta-analysis of the odds ratios of association between rs7903146 polymorphism of *TCF7L2 *gene and T2DM for T homozygotes vs. C homozygotes**.

**Figure 6:**
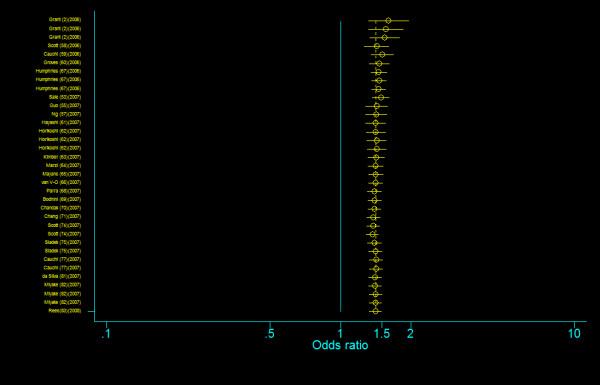
**Cumulative meta-analysis of the odds ratios of association between rs7903146 polymorphism of *TCF7L2 *gene and T2DM for heterozygotes vs. C homozygotes**.

We did not find any evidence of publication bias of the eligible studies. Funnel plots for the comparisons most commonly made, that is, for T allele vs. C allele, T homozygotes (TT) vs. C homozygotes (CC), and heterozygotes (TC) vs. C homozygotes (CC) are indicated in figure 1, 2 and 3. These three plots give corrected *P *= 0.410 (corrected *z *= 0.82), corrected *P *= 0.379 (corrected *z *= 0.88), and corrected *P *= 0.426 (corrected *z *= 0.80), respectively. Figure 4, 5, and 6 indicate the cumulative meta-analysis after each study from March 2006 to February 2008, for T allele vs. C allele, T homozygotes vs. C homozygotes, and heterozygotes vs. C homozygotes. The random-effects odds ratio of T homozygotes vs. C homozygotes waved slightly among studies performed in 2006 and has changed very little from around 2.0 after the eleventh study (figure 5). Cumulative meta-analysis of the other two comparisons by year of publication showed that the association between T2DM and *TCF7L2 *IVS3C>T polymorphism has remained significant and been consistent over time (figure 4 and figure 6).

#### Allele comparisons

**Figure 7:**
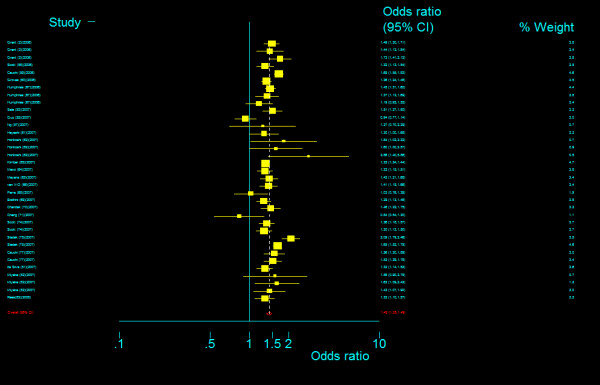
**Odds ratios of association between rs7903146 polymorphism of *TCF7L2 *gene and T2DM for T allele vs. C allele**.

Data from control groups were used for calculating the summary allele frequency. The frequency of the risk allele T at *TCF7L2 *gene among controls was 20.2 percent (95% confidence interval (*CI*): 16.3, 24.1) and was considerable variation in different ethnic populations (for Caucasians, 29.0% (95% *CI*: 28.3, 29.7); for North Europeans, 21.9% (95% *CI*: 16.2, 27.6); for East Asians, 3.2 (95% *CI*: 2.6, 3.9); for Indians, 28.1% (95% *CI*: 25.9, 30.3); for others, 23.0% (95% *CI*: 9.4, 36.6)). We first contained all the 35 studies to evaluate the overall association between *TCF7L2 *IVS3C>T polymorphism and T2DM. With our allele-based analysis, the pooled odds ratio suggested a 1.416-fold increase in susceptibility to T2DM among persons with the T allele (random-effects *OR*_TvC _= 1.416, 95% *CI*: 1.346, 1.491), with a finding that was highly statistically significant (*P *= 0.000), but there was significant between-study heterogeneity (*Q *= 104.966, *P *= 0.000; *I*^2 ^= 67.6%) (Table 6 [Additional file 1], figure 7).

#### Genotype comparisons

**Figure 8:**
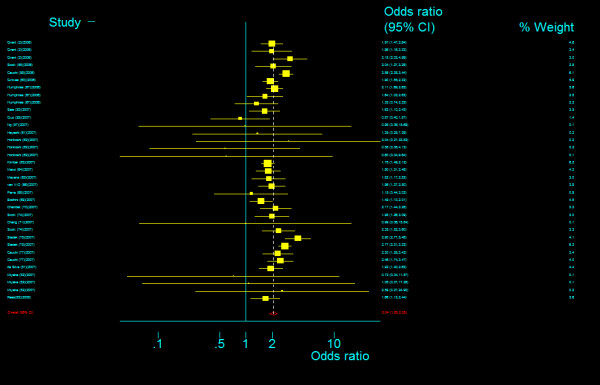
**Odds ratios of association between rs7903146 polymorphism of *TCF7L2 *gene and T2DM for T homozygotes vs. C homozygotes**.

**Figure 9:**
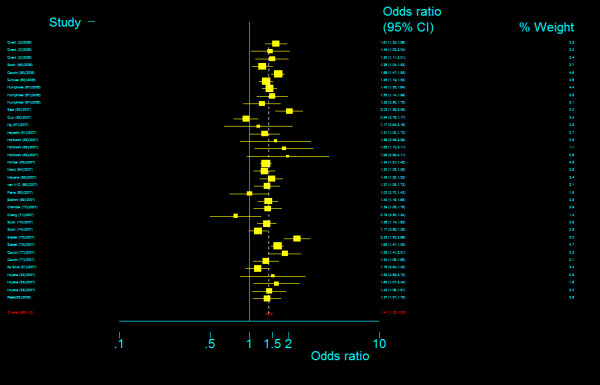
**Odds ratios of association between rs7903146 polymorphism of *TCF7L2 *gene and T2DM for heterozygotes vs. C homozygotes**.

The genotype frequency of *TCF7L2 *IVS3C>T polymorphism between case and control groups is characterized in web table. The genotype effects for TT versus CC (*OR*_1_) and TC versus CC (*OR*_2_) were calculated for each study (data are not shown and available from the first author on request). In our primary concern, multivariate meta-analysis was conducted to estimate the pooled effects and there was an significant increased risk of T2DM among populations with both homozygous variant TT genotype (Bayesian random-effect *OR*_1 _= 1.968, 95 percent credible interval (*CrI*): 1.790, 2.157) and heterozygous variant TC genotype (Bayesian random-effect *OR*_2 _= 1.406, 95 percent *CrI*: 1.341, 1.476), with a "moderate" between-study heterogeneity (*Q *= 66.102, *P *= 0.001, *I*^2 ^= 48.6%, figure 8) in the former and a "notable" between-study heterogeneity (*Q *= 84.028, *P *= 0.000, *I*^2 ^= 59.5%, figure 9) in the latter. The estimated stochastic parameter λ_1 _was 0.504 (95 percent *CrI*: 0.461, 0.551), which suggested a codominant genetic mode of action.

#### Examination of heterogeneity

Meta-regression was used to explore the source of heterogeneity and it was found that ethnicity was the only co-variable associated with both ln*OR*_1 _(meta-regression beta coefficient (β) = -0.0993, *P *= 0.002) and ln*OR*_2 _(β = -0.0360, *P *= 0.002). We then did a subgroup analysis by stratifying study samples into five ethnic groups, that is, Caucasians (11 samples), North European (6 samples), East Asian (9 samples), Indians (4 samples) and other racial descent group (5 samples), and found that this stratification can remove all the between-study heterogeneity among North Europeans, East Asians and Indians for both *OR*_1 _and *OR*_2_, rather than that among Caucasians and other racial group (Table 6, Additional file 1). We then did a further subgroup analysis of Caucasians by stratifying them into French Caucasians versus other Caucasians and extracted the studies with African populations for an independent analysis, and found that such analyses can only remove heterogeneity among these three populations for *OR*_1 _rather than *OR*_2 _(Table 6, Additional file 1). The pooled *OR*_1 _for six ethnic groups by descending ranking, that is, French Caucasians, other Caucasians, North Europeans, Africans, Indians, and East Asians, was 3.051(95%*CrI*: 2.039, 4.906), 1.989 (95%*CrI*: 1.807, 2.216), 1.944 (95%*CrI*: 1.639, 2.284), 1.802 (95%*CrI*: 1.292, 2.480), 1.669 (95%*CrI*: 1.373. 2.104), and 1.595 (95%*CrI*: 0.537, 2.653), respectively, and the pooled *OR*_2 _by descending ranking was 1.766 (95%*CrI*: 1.427, 2.278) for French Caucasians, 1.696 (95%*CrI*:1.258, 2.242) for Africans, 1.406 (95%*CrI*: 1.230, 1.617) for Indians, 1.357 (95%*CrI*: 1.255, 1.474) for North Europeans, 1.352 (95%*CrI*: 1.160, 1.591) for East Asians, and 1.349 (95%*CrI*: 1.278, 1.431) for other Caucasians (Table 2, Additional file 1). The estimated parameter λ_1 _by descending ranking was 0.981 (95 percent *CrI*: 0.759, 0.999) for Africans, 0.676 (95 percent *CrI*: 0.452, 0.957) for Indians, 0.670 (95 percent *CrI*: 0.278, 0.995) for East Asians, 0.511 (95 percent *CrI*: 0.449, 0.578) for French Caucasians, 0.472 (95 percent *CrI*: 0.345, 0.624) for North Europeans, and 0.436 (95 percent *CrI*: 0.366, 0.511) for other Caucasians, which suggested codominant genetic mode of action among these different ethnic populations except for Africans where dominant mode of action was found.

Although BMI was not associated with both ln*OR*_1 _and ln*OR*_2 _by separate meta-regression analysis, the significant association was found when we evaluate it together with ethnicity (for ln*OR*_1_, β = -0.0512, *P *= 0.038; for ln*OR*_2_, β = -0.0311, *P *= 0.004). In addition, some references reported that BMI may interact with *TCF7L2 *gene to increase the risk of T2MD. Therefore, we did a subgroup analysis for BMI (normal (< 30.0 kg/m^2^) vs. abnormal (≥ 30.0 kg/m^2^). However, such stratification cannot remove heterogeneity for both ln*OR*_1 _(*Q *= 35.017, *P *= 0.006, *I*^2 ^= 52.1%) and ln*OR*_2 _(*Q *= 28.046, *P *= 0.013, *I*^2 ^= 50.0%). The pooled *OR*_1_, *OR*_2_, and λ_1 _for populations with normal BMI were 2.017 (95%*CrI*: 1.711, 2.347), 1.470 (95%*CrI*: 1.350, 1.609), and 0.554 (95%*CrI*: 0.481, 0.637), respectively. The pooled *OR*_1_, *OR*_2_, and λ_1 _for populations with abnormal BMI were 1.931 (95%*CrI*: 1.670, 2.213), 1.337 (95%*CrI*: 1.250, 1.431), and 0.441 (95%*CrI*: 0.387, 0.500), respectively. Logistic regression analysis indicated that there was no significant difference between populations with normal and abnormal BMI for both ln*OR*_1 _(*P *= 0.855) and ln*OR*_2 _(*P *= 0.716).

### IVS4G>T

Our searches identified 29 studies (totally 57,235 participants; 28,188 cases of T2DM, 29,047 controls) meeting our inclusion criteria. All of these studies examined the IVS4G>T polymorphism (Table 4, Additional file 1). Allele and genotype data were available for all the cases and controls in the eligible studies. There was no evidence of Hard-Weinberg disequilibrium in both cases and controls.

#### Bias diagnostics

**Figure 10:**
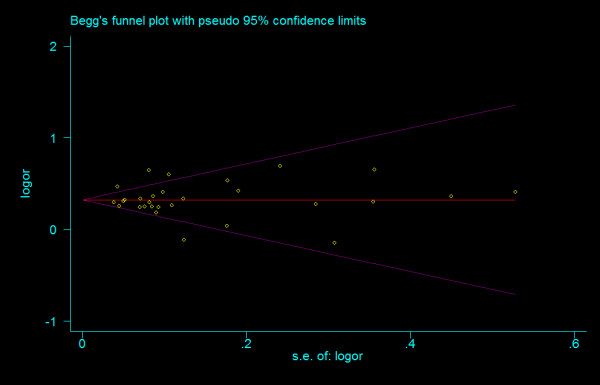
**Funnel plots of rs12255372 polymorphism studies for T allele vs. G allele (corrected *z *= 0.96, corrected *P *= 0.339)**.

**Figure 11:**
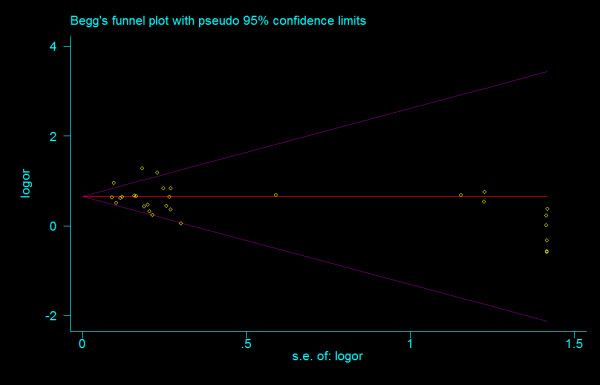
**Funnel plots of rs12255372 polymorphism studies for T homozygotes vs. G homozygotes (corrected *z *= 0.62, corrected *P *= 0.536)**.

**Figure 12:**
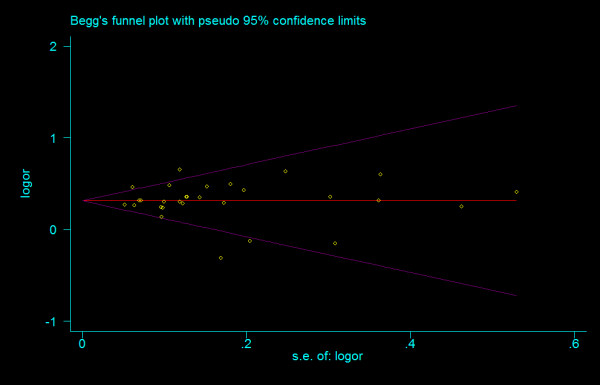
**Funnel plots of rs12255372 polymorphism studies for heterozygotes vs. G homozygotes (corrected *z *= 0.77, corrected *P *= 0.442)**.

**Figure 13:**
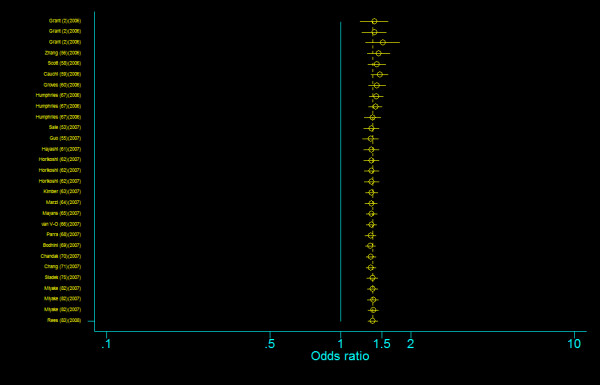
**Cumulative meta-analysis of the odds ratios of association between rs12255372 polymorphism of *TCF7L2 *gene and T2DM for T allele vs. H allele**.

**Figure 14:**
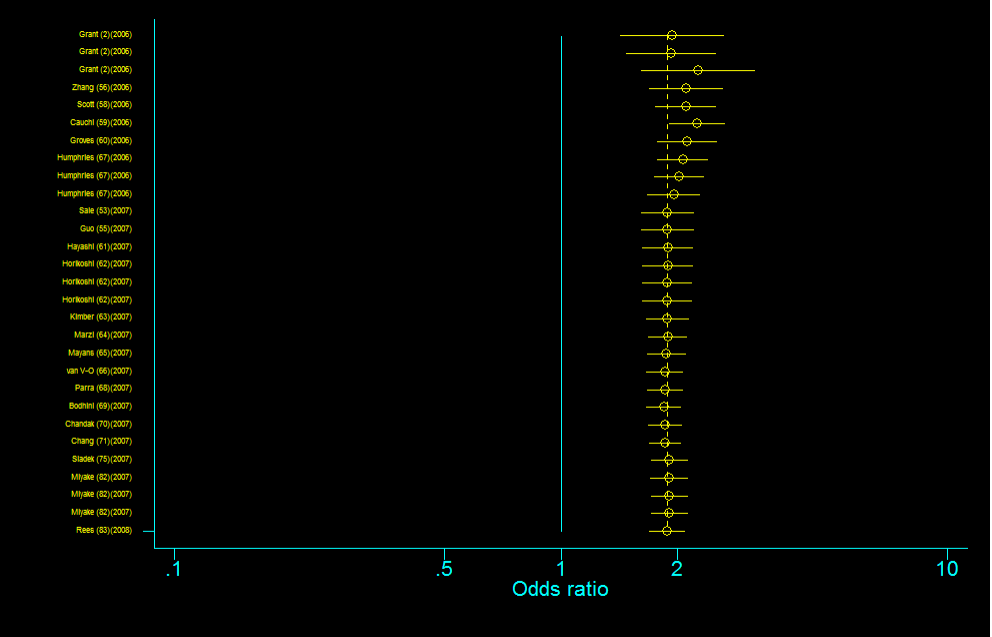
**Cumulative meta-analysis of the odds ratios of association between rs12255372 polymorphism of *TCF7L2 *gene and T2DM for T homozygotes vs. G homozygotes**.

**Figure 15:**
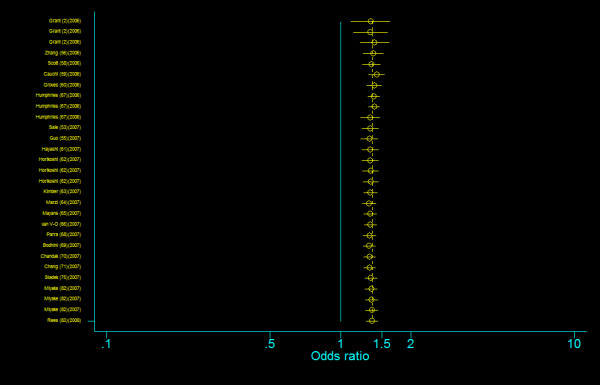
**Cumulative meta-analysis of the odds ratios of association between rs12255372 polymorphism of *TCF7L2 *gene and T2DM for heterozygotes vs. G homozygotes**.

There was no evidence of small study bias and publication bias for any comparisons. Funnel plots for the comparisons made for T allele vs. G allele, T homozygotes (TT) vs. G homozygotes (GG), and heterozygotes (TG) vs. G homozygotes are shown in figure 10, 11 and 12. These three funnel plots give corrected *P *= 0.339 (corrected *z *= 0.96), corrected *P *= 0.536 (corrected *z *= 0.62), and corrected *P *= 0.442 (corrected *z *= 0.77), respectively. Figure 13, 14 and 15 indicate the cumulative meta-analysis after each study from March 2006 to February 2008, for allele-based analysis and two genotype-based models of IVS4G>T polymorphism. The random-effects odds ratio of allele-based analysis was significant more than 1 and changed very little from around 1.379 after the first study (figure 13). The random-effects odds ratio of T homozygotes vs. G homozygotes waved slightly among studies performed in 2006 and has changed very little from around 1.884 after the eleventh study (figure 14). Cumulative meta-analysis of heterozygotes vs. G homozygotes by year of publication showed that the association between T2DM and *TCF7L2 *IVS4G>T polymorphism has remained significant and been consistent over time (figure 15).

#### Allele comparisons

**Figure 16:**
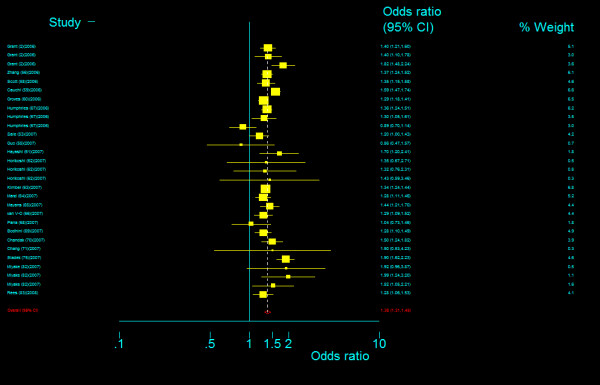
**Odds ratios of association between rs12255372 polymorphism of *TCF7L2 *gene and T2DM for T allele vs. H allele**.

The frequency of the risk allele T at *TCF7L2 *gene among controls was 18.0 percent (95% *CI*: 13.5, 22.5) and was considerable variation in different ethnic populations (for Caucasians, 27.9% (95% *CI*: 27.0, 28.9); for North Europeans, 22.3% (95% *CI*: 12.6, 32.1); for East Asians, 2.1 (95% *CI*: 1.4, 2.7); and for Indians, 23.1% (95% *CI*: 17.8, 28.3)). We first contained all the 29 studies to evaluate the overall association between *TCF7L2 *IVS4G>T polymorphism and T2DM. With our allele-based analysis, the summary odds ratio suggested a 1.379-fold increase in susceptibility to T2DM among persons with the T allele (random-effects *OR*_TvG _= 1.379, 95% *CI*: 1.307, 1.454), with a finding that was highly statistically significant (*P *= 0.000), but there was significant between-study heterogeneity (*Q *= 66.191, *P *= 0.000; *I*^2 ^= 57.7%) (Table 6 [Additional file 1], figure 16).

#### Genotype comparisons

**Figure 17:**
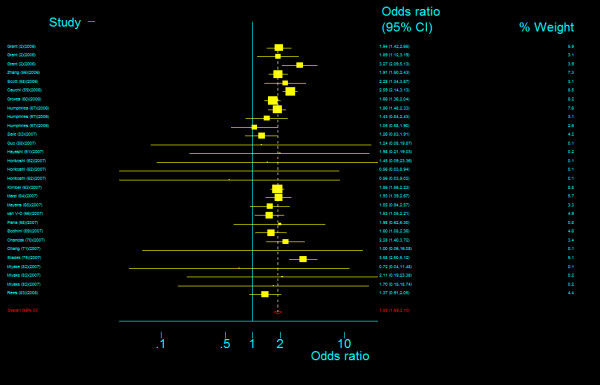
**Odds ratios of association between rs12255372 polymorphism of *TCF7L2 *gene and T2DM for T homozygotes vs. G homozygotes**.

**Figure 18:**
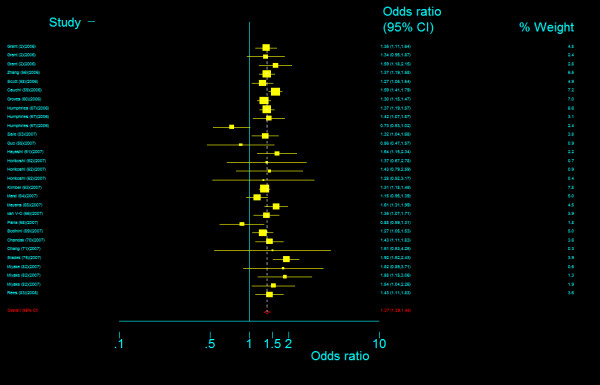
**Odds ratios of association between rs12255372 polymorphism of *TCF7L2 *gene and T2DM for heterozygotes vs. G homozygotes**.

The genotype frequency of *TCF7L2 *IVS4G>T polymorphism between case and control groups is characterized in Table 4, Additional file 1. The genotype effects for TT versus GG (*OR*_3_) and TG versus GG (*OR*_4_) were calculated for each study (data are not shown and available from the first author on request). In our primary concern, multivariate meta-analysis was conducted to estimate the pooled effects and there was an significant increased risk of T2DM among populations with both homozygous variant TT genotype (Bayesian random-effect *OR*_2 _= 1.885, 95 percent credible interval (*CrI*): 1.698, 2.088) and heterozygous variant TG genotype (Bayesian random-effect *OR*_4 _= 1.360, 95 percent *CrI*: 1.291, 1.433), with a "moderate" between-study heterogeneity both in the former (*Q *= 46.515, *P *= 0.015, *I*^2 ^= 39.8%, figure 17) and in the latter (*Q *= 47.980, *P *= 0.011, *I*^2 ^= 41.6%, figure 18). The estimated stochastic parameter λ_2 _was 0.486 (95 percent *CrI*: 0.431, 0.545), which suggested a codominant genetic mode of action.

#### Examination of heterogeneity

Meta-regression was used to explore the source of heterogeneity and it was found that ethnicity was the only co-variable associated with both ln*OR*_3 _(β = -0.1083, *P *= 0.005) and ln*OR*_4 _(β = -0.0418, *P *= 0.047). We then did a subgroup analysis by stratifying study samples into five racial descent groups, that is, Caucasians (9 samples), North European (4 samples), East Asian (8 samples), Indians (4 samples) and other racial descent group (4 samples), and found that this stratification can remove all the between-study heterogeneity among North Europeans, East Asians and Indians for both *OR*_3 _and *OR*_4_, rather than that among Caucasians and other ethnic group (Table 6, Additional file 1). We then extracted the studies with other Caucasians (7 studies) for an independent analysis, and found that such analysis can well-done remove the heterogeneity among these studies for both *OR*_3 _(*Q *= 8.407, *P *= 0.210, *I*^2 ^= 28.6%) and *OR*_4 _(*Q *= 4.327, *P *= 0.633, *I*^2 ^= 0.0%) (Table 6, Additional file 1). We did not do a further analysis of other ethnic group because of different origin of populations in these studies. The pooled *OR*_3 _for four ethnic groups (data of other ethnic group not shown for the heterogeneity of these studies) by descending ranking, that is, Caucasians, North Europeans, East Asians and Indians, was 2.091 (95%*CrI*: 1.755, 2.528; for other Caucasians, *OR*_3 _= 1.860 (95%*CrI*: 1.672, 2.012)), 1.889 (95%*CrI*: 1.485, 2.412), 1.875 (95%*CrI*: 0.464~3.439), and 1.593 (95%*CrI*: 1.286, 2.086), respectively, and the pooled *OR*_4 _by descending ranking was 1.569 (95%*CrI*: 1.304, 1.887) for East Asians, 1.413 (95%*CrI*: 1.299, 1.555; for other Caucasians, *OR*_4 _= 1.326 (95%*CrI*: 1.251, 1.412)) for Caucasians, 1.380 (95%*CrI*: 1.222, 1.569) for North Europeans, and 1.358 (95%*CrI*: 1.176, 1.579) for Indians (Table 6, Additional file 1). The estimated parameter λ_2 _by descending ranking was 0.744 (95 percent *CrI*: 0.341, 1.996) for East Asians, 0.657 (95 percent *CrI*: 0.403, 0.982) for Indians, 0.507 (95 percent *CrI*: 0.347, 0.748) for North Europeans, and 0.470 (95 percent *CrI*: 0.410, 0.534) for Caucasians (for other Caucasians, λ_2 _= 0.455, 95 percent *CrI*: 0.376, 0.541), which suggested codominant genetic mode of action among all these four ethnic populations.

Although BMI was not associated with both ln*OR*_3 _and ln*OR*_4 _by separate meta-regression analysis, the significant association was found when we evaluate it together with racial descent (for ln*OR*_3_, β = -0.0600, *P *= 0.029; for ln*OR*_4_, β = -0.0467, *P *= 0.000). In addition, some references reported that BMI may interact with *TCF7L2 *gene to increase the risk of T2MD. Therefore, we also did a subgroup analysis for BMI in this polymorphism. However, such stratification can remove few heterogeneity for both ln*OR*_3 _(*Q *= 22.517, *P *= 0.084, *I*^2 ^= 37.2%) and ln*OR*_4 _(*Q *= 19.733, *P *= 0.042, *I*^2 ^= 47.0%). The pooled *OR*_3_, *OR*_4_, and λ_2 _for populations with normal BMI were 1.795 (95%*CrI*: 1.480, 2.211), 1.397 (95%*CrI*: 1.254, 1.567), and 0.572 (95%*CrI*: 0.448, 0.731), respectively. The pooled *OR*_3_, *OR*_4_, and λ_2 _for populations with abnormal BMI were 1.957 (95%*CrI*: 1.710, 2.210), 1.343 (95%*CrI*: 1.261, 1.429), and 0.442 (95%*CrI*: 0.377, 0.511), respectively. Logistic regression analysis indicated that there was no significant difference between populations with normal and abnormal BMI for both ln*OR*_1 _(*P *= 0.069) and ln*OR*_2 _(*P *= 0.152).

### Other polymorphisms

The C allele of IVS3T>C polymorphism (10 studies with 15,718 participants (8,175 cases of T2DM, 7,543 controls); random-effects *OR*_CvT _= 1.323, 95% *CI*: 1.232, 1.419, *P *= 0.000; *Q *= 12.615, *P *= 0.181, *I*^2 ^= 28.7 percent) and the C allele of IVS4G>C polymorphism (15 studies with 21,161 participants (11,260 cases of T2DM, 9,901 controls); random-effects *OR*_CvG _= 1.238, 95 percent *CI*: 1.153, 1.330, *P *= 0.000; *Q *= 20.968, *P *= 0.102, *I*^2 ^= 33.2 percent) were significantly associated with T2DM (Table 6, Additional file 1). The pooled *OR*_5_, *OR*_6_, and λ_3 _for IVS3T>C polymorphism were 1.771 (95%*CrI*: 1.525, 2.062), 1.292 (95%*CrI*: 1.199, 1.402), and 0.450 (95%*CrI*: 0.330, 0.598), respectively. The pooled *OR*_7_, *OR*_8_, and λ_4 _for IVS4G>C polymorphism were 1.471 (95%*CrI*: 1.300, 1.706), 1.242 (95%*CrI*: 1.152, 1.355), and 0.561(95%*CrI*: 0.393, 0.777), respectively. These all suggested a codominant genetic mode of action among such two polymorphisms. We did not find any evidence of small sample bias and publication bias for any of the studies (IVS3T>C polymorphism: for *OR*_5_, corrected *z *= 0.54, corrected *P *= 0.592; for *OR*_6_, corrected *z *= 1.43, corrected *P *= 0.152; IVS4G>C polymorphism: for *OR*_7_, corrected *z *= 0.40, corrected *P *= 0.692, for *OR*_8_, corrected *z *= 1.39, corrected *P *= 0.166), and cumulative meta-analysis results for both allele-based and genotype-based analysis of the above two polymorphisms were stable (data and figure are not shown and available from the first author on request).

## Discussion

### Main findings

This is a large meta-analysis, including data from 25 papers involved in 36 genetic association studies with exactly 35,843 cases of T2DM and 39,123 controls, carefully avoiding the double-counting of participants in the study. The HuGE systematic review provides the most recent and comprehensive evaluation of the association between four *TCF7L2 *gene polymorphisms and susceptibility to T2DM. We find that near 70,000 subjects (33,135 cases of T2DM and 36,316 controls) were from 35 studies concerning the IVS3C>T polymorphism, and over 55,000 (28,188 cases of T2DM and 29,047 controls) subjects were from 29 studies investigating the IVS4G>T polymorphism. These two variants were the main study focus on the association between *TCF7L2 *gene polymorphisms and susceptibility to T2DM.

On one hand, the results indicates notable associations between two main *TCF7L2 *gene polymorphisms, namely, IVS3C>T as well as IVS4G>T, and T2DM. The magnitudes of this association were moderate, however, statistically significant. Our primary analysis finds that among IVS3C>T polymorphism, TC heterozygotes carry just over a 1.4-fold increased risk of T2MD, and TT homozygous variants carry near a 2.0-fold increase in T2MD risk when compared with CC homozygotes, and that among IVS4G>T polymorphism, TG heterozygotes carry near a 1.4-fold increased risk of T2MD, and TT homozygous variants carry approximate a 1.9-fold increase in T2MD risk when compared with GG homozygotes. Significant magnitudes of genetic effect for heterozygotes of these two variants were confirmed by the analysis on the pooled odds ratios among different ethnic groups with slight differences except for East Asians where higher pooled odds ratio was observed in IVS4G>T polymorphism when compared with that of the other ethnic populations, and genetic effect sizes for homozygotes of these two variants were also strengthened by the analysis on the pooled odds ratios among different ethnic groups including Caucasians, North Europeans, Africans and Indians by showing a small variation. However, we found that there was no significant association between such two homozygotes and disease among East Asians, and therefore, conflicting results have appeared, in which studies on Chinese provided negative results [57,71], however, positive results were observed among Japanese populations [61,62,82]. Our meta-analysis on 9 studies of IVS3C>T polymorphism and 7 studies of IVS4G>T polymorphism among East Asians indicates a moderate statistical association and shows that the heterozygous variants contributed all the increased risk of T2MD among this population. This distinct disparity may suggest different mechanism of gene-disease between East Asians and other ethnic population. Moreover, although less samples and subjects were provided, the IVS3T>C and IVS4G>C polymorphisms are also significantly associated with T2DM. We then suggest that *TCF7L2 *is the most common susceptible gene for T2DM among various ethnic groups in the world.

On the other hand, we made no assumptions about genetic models and finds that the pooled odds ratios can clearly fit a multiplicative model and the lambda (λ) parameter can well-done indicate a codominant genetic mode of action with tight *CI*s among all these four *TCF7L2 *gene polymorphisms. We also explored the genetic mode among different ethnic populations and similar results were observed in Caucasians, North Europeans, East Asians and Indians except for Africans, where dominant genetic mode is suggested for IVS3C>T polymorphism. We cannot verify the genetic mode of action for Africans in the other three polymorphisms due to few studies. In addition, genetic mode of IVS3C>T variant among Africans also need a further verification by more studies. Even so, we nevertheless suggest a potential multiplicative genetic model for the four *TCF7L2 *gene polymorphisms.

The effect size of overall association between IVS3C>T variant and T2MD is near to 1.42, which is lower than that in a former meta-analysis about *TCF7L2 *and T2MD performed by Cauchi et al [77], and larger than that in a pooled analysis of three genome-wide association (GWA) study [73,74,76]. we supplement some studies about East Asians and Pima Indians [55,57,71,88] in which risk homozygous variant TT was scanty, and excluded several studies without genotype frequency data, which may decrease the overall effect but provide a more comprehensive understanding of the association. Even so, we cannot find any significant difference of effect size between our study with the former meta-analysis as well as the pooled GWA studies. In addition, we computed the PAR of *TCF7L2 *according to IVS3C>T variant and the PAR for the combined genotypes TT and TC were 16.9, 23.2, 14.1, 2.5, 17.9, 27.0 for overall, Caucasians, North Europeans, East Asians, Indians, and Africans, respectively, suggesting this gene polymorphism may contribute near 1/5 of all T2MD in the globe except for East Asians.

Our findings were based on some gene-association studies and tens of thousands participants and were robust to each of the planned sensitivity analyses used. We cannot find any evidence of publication bias and small study bias by funnel plots and cumulative meta-analysis, but, considerable between-study heterogeneity was found. Between-study heterogeneity may be due to differences in sample selection (e.g., in age, sex, diagnosis, sample content, etc), or in methods (e.g., genotyping method), or it may be due to real differences in populations (e.g., in race), or in interactions with other risk factors (genetic or/and environmental factors). The results of primary analysis (genotype-based) on the four polymorphisms showed statistically significant between-study heterogeneity for both IVS3C>T polymorphism (*OR*_1 _and *OR*_2_), and IVS4G>C polymorphism (*OR*_3 _and *OR*_4_). In this Human Genome Epidemiology review, we examined five potential sources of between-study heterogeneity for genotype-based model by meta-regression and it was suggested that ethnicity was the only co-variable associated with the four *OR*s. In fact, more detailed manner of ethnic stratification, namely, French Caucasians, other Caucasians, North Europeans, East Asians, Indians, and Africans, can remove near all the heterogeneity of studies about both IVS3C>T and IVS4G>C polymorphisms for *OR*_1 _to *OR*_4_. Study results reported by several articles showed that BMI may cooperate with the *TCF7L2 *gene to increase the risk of the T2DM. However, disagreement of the results was published by different researchers. Cauchi et al[59], Horikoshi et al [62] and Humphries et al [67] separated to analyze the nonobese type 2 diabetic subjects (BMI<30 Kg/m^2^) and more higher allelic *OR*s or *RR*s were obtained in IVS3C>T and IVS4G>C polymorphisms; whereas, Miyake et al [82], Chandak et al[70], and Dahlgren et al [104] found that there was a slight increase or no change of odds ratio by adjustment of BMI. So we stratified the studied by BMI (normal vs. abnormal) and found that significant between-study heterogeneity nevertheless appeared in all the two subgroups. Both studies on IVS3C>T and IVS4G>C polymorphisms provided us for the similar results. But, we found a significant association when it was evaluated together with ethnicity. These results, on the one hand, suggested that BMI may be a possible factor to impact on the effect of *TCF7L2 *gene on T2DM by a indirect action; on the other hand, were difficult for us to explain the deep-seated reasons that how the BMI influence the effect of *TCF7L2 *gene on T2DM because we cannot gain the original data of BMI in each article. This is a general limitation of meta-analysis. So we hope the reasonable interpretation will be presented in the subsequent studies. Although sex [56,63,68,71,105], age of cases [63,68,71,105], drugs[106], constitution[64,69], and lifestyle [64,69,105] may regulate the effects of *TCF7L2 *gene, we cannot test the effects of age, sex, drugs and lifestyle on heterogeneity due to without sufficient related data being used. In addition, the age of the control NGT groups may be associated with the heterogeneity and the young controls may develop diabetes at a later date. However, there are also insufficient data.

### Potential gene-gene and gene-environment interaction

T2DM seems to result from a complicated interplay of genetic and environmental factors influencing a number of intermediate traits of relevance to the diabetic phenotype (e.g., insulin secretion, insulin action, fat distribution, obesity). As a matter of fact, T2DM appears to be composed of subtypes where genetic susceptibility is strongly associated with environmental factors at one end of the spectrum, and highly genetic forms at the other end. Thus, there are several possible interactions between gene and gene or between gene and environmental factors.

Recent years, a series of new loci in some genes have been identified to contribute about 10~30% population attributable risk (PAR) of T2DM [73-76,107]. But, the overall PAR seems to far less than the cumulative effect. So there must be some potential interactions among genes. The strongest known T2DM association (random-effects *OR*_TvC_≈1.42) was recently mapped to the transcription factor *TCF7L2*, a gene that is a target of the Wnt signaling pathway [50]. Whereafter, some other genes such as *HHEX*, *IDE*, *DKK3 *and *KIF11*, which are also in the Wnt signaling pathway [108-113] may have some interactions with *TCF7L2 *on the risk of T2DM. Cauchi et al [59]found that the expression of *TCF7L2 *gene was downregulated in obese subjects developing T2DM and they then brought forward a hypothesis of a potential interaction between *TCF7L2 *and *Calpain-10 *by a supporting material [114] that suggested that the Calpain system was involved in the constitutive regulation of β-catenin signaling functions. Wnt signaling has recently been shown to regulate pancreatic β-cell proliferation and the author suggested a possible interation between *TCF7L2 *gene and β-catenin gene [115]. However, there is not any reported publication about the interaction between *TCF7L2 *other related genes. These hypothetical interactions and their specific effects on T2DM, as well as functional analyses, will be required to further elucidate the role of variation in *TCF7L2 *in the pathogenesis of T2MD and very large samples are needed.

A variety of environmental factors can be implicated in the phenotype of T2DM, such as obesity, hypertension, bad lifestyle (smoking, drinking, high-energy diet), short of exercise, malnutrition, and some drugs [116]. All the above environmental factors may interact with the *TCF7L2 *gene to influence T2DM. So far, few specific gene-environment interactions have been described for *TCF7L2 *gene polymorphisms. Because obesity is a major determinant of development of T2DM, most patients with T2DM are obese when they develop diabetes, and obesity aggravates the insulin resistance. BMI and waist circumstance may become the main focus on attention of interactions, particularly in studies of IVS3C>T and IVS4G>C polymorphism among T2DM. Cauchi and colleagues [117] reported that IVS3C>T risk allele is more prevalent in non-obese than in obese diabetics. Humphries et al [67] found a statistically significant interaction between IVS3C>T as well as IVS4G>C genotype and BMI. Wang et al [118] replicated the result of Humphries et al. In succession, Helgason et al [47] discovered that two haplotypes, that is, HapA and HapB_T2D _had a interaction with BMI, and that the former was associated with increased BMI, whereas the latter was associated decreased BMI. Florez et al [106], observed that another index of obesity, waist circumstance, showed a nominally significant interation with both IVS3C>T and IVS4G>T in spite of no interaction between BMI and genotype. In addition, plasma triglyceride (TG) and C-reactive protein (CRP) have also been found to interact with genotype of both IVS3C>T and IVS4G>T [67]. These early findings suggestive of gene-environment interactions with the different *TCF7L2 *gene polymorphisms should be interpreted extremely cautiously, however, and much larger and more detailed studies are looked forward to substantiating such putative interactions with appropriate power and rigor. Complementarily, Smoking and drinking were important risk factors to influence on T2DM and they can also interact with genetic factor to increase the risk of this disease [116]. We found a higher proportion of current or/and past smokers and drinkers in the study populations. But, it was regretted for us that there was no any report about the interactions between *TCF7L2 *gene polymorphisms and smoking or/and drinking in all the open publications. This is a meaningful and valuable domain to explore.

### Limitation of this meta-analysis

The lack of information especially the genotype data from some articles was the main limitation, we have done our possible to contact exhaustibly to the authors of publication that did not provide the original data and obtained full data on about 75 percent of subjects involved in various ethnic populations. Next, the quality of diagnosis, match, and genotyping varied a lot among studies, but the summary results were not changed when these questions were discussed carefully. There was considerable heterogeneity in IVS3C>T and IVS4G>T polymorphisms in our initial results. However, we discovered the main sources of the heterogeneity and were able to remove it finally. A question we cannot resolve is that how the four snps discussed here in strong LD will lead to differences in T2DM association among diverse populations. Besides, since environment factors and genes, such as obesity, smoking, drinking, and hypertension as well as plasma TG, and gene related to the Wnt signaling pathway are strong candidates for a potential interaction with *TCF7L2 *gene polymorphisms, the lack of data available to us on most of those environmental factors (except for obesity) and genes was a limitation, which we hope will be demonstrated by the following studies.

## Conclusions and recommendation for future researches

This large meta-analysis summarizes the strong evidence for an association between *TCF7L2 *gene and T2DM both overall and in Caucasians, North Europeans, East Asians, Indians, and Africans, and suggested a potential multiplicative genetic model for all the four polymorphisms of *TCF7L2 *gene among different ethnic populations except for Africans, where additive genetic mode is suggested for IVS3C>T polymorphism. Our study results also suggest that IVS3C>T and IVS4G>T variants of *TCF7L2 *gene can be taken as reference loci for exploring T2DM susceptibility. We found and removed the main source of heterogeneity; however, we cannot find any evidence of bias. Furthermore, we estimated the potential gene-gene and gene-environmental interactions by which common variants in the *TCF7L2 *gene influence risk of T2DM. These two domains are just the recommendation for future researches.

## Abbreviations

HuGE: Human Genome Epidemiology; T2DM: type 2 diabetes mellitus; TCF7L2: Transcription factor 7-like 2; CI: confidence interval; CrI: credible interval; OR: odds ratio; NGT: normal glucose tolerant; BMI: body mass index; HWE: Hardy-Weinberg equilibrium.

## Competing interests

The authors declare that they have no competing interests.

## Authors' contributions

BZ, YT, YL, and YZ carried out the design of this meta-analysis, analyzed the data and drafted the manuscript. YZ and JYY conducted a searching, data extraction, and statistical analysis on the manuscript. YWZ performed a searching and data extraction on the manuscript. HCL participated in designing and writing for the manuscript. All authors read and approved the final manuscript.

## Pre-publication history

The pre-publication history for this paper can be accessed here:



## Supplementary Material

Additional file 1 **Title: Tables. Tables 1 – 6.** Click here for file
