# Supplementary material for: Association between TCF7L2 gene polymorphisms and susceptibility to Type 2 Diabetes Mellitus: a large Human Genome Epidemiology (HuGE) review and meta-analysis
Source: BMC Med Genet. 2009 Feb 19;10:15. doi: 10.1186/1471-2350-10-15 (PMC2653476; doi:10.1186/1471-2350-10-15)
Supplement: Additional file 1 — Title: Tables. Tables 1 – 6. [file 1471-2350-10-15-S1.doc]

**Table 1. Characteristics of case-control studies included in a meta-analysis of the association between the *TCF7L2* gene polymorphisms and type 2 diabetes mellitus**

| First author  (Ref. No.) | Year | Country(ies) | Ethnicity | Sex  Composition (male) | | Mean study age (year) | |  | BMI  (kg/m^2^) | |  | Characteristics/selection of cases and controls | |  | No. of eligible subjects | |
| --- | --- | --- | --- | --- | --- | --- | --- | --- | --- | --- | --- | --- | --- | --- | --- | --- |
|  |  |  |  |  |  | Cases | Controls |  | Cases | Controls |  | Cases | Controls |  | Cases | Controls |
| Grant | 2006 | Iceland | North European | 699 | 473 | 66.7 | 50.7 |  | 29.7 | 26.7 |  | Type 2 diabetic patients | Nondiabetic controls |  | 1185 | 931 |
| Grant | 2006 | Denmark | North European | 0 | 0 | 73.7 | 72.0 |  | 28.1 | 25.7 |  | Type 2 diabetic patients from PERF study in Denmark | Nondiabetic controls |  | 228 | 539 |
| Grant | 2006 | USA | Caucasian | 250 | 328 | 63.3 | 60.0 |  | 29.7 | 28.2 |  | Type 2 diabetic patients from PENN CATH study in USA | Nondiabetic controls |  | 361 | 530 |
| Sale | 2007 | USA | African | 225 | 292 | 62.2 | 49.3 |  | NR | NR |  | Unrelated patients with type 2 diabetes and excluded type 1 diabetes | Regional matched controls without type 2 diabetes |  | 577 | 596 |
| Guo | 2007 | USA | American Pima Indian | 580 | 902 | 37.2 | 31.1 |  | 33.4 |  |  | Type 2 diabetes subjects according to the WHO criteria | Racial matched nondiabetic controls |  | 1561 | 1940 |
| Zhang | 2006 | USA | Caucasian | 886 | 896 | 56.6 | 56.5 |  | 30.6/27.7 | 27.4  27.7 |  | Self-reported diabetes patients confirmed by a validated supplementary questionnaire. | Age, race, and BMI matched controls without type 2 diabetes |  | 1617 | 1992 |
| Ng ¶ | 2007 | China | East Asian | 164 | 168 | 39.4 | 40.6 |  | 25.8 | 22.5 |  | Unrelated patients with type 2 diabetes | Nondiabetic controls |  | 433 | 419 |
| Scott | 2006 | Finland | North European | 645 | 518 | 64.3/61.0 | 69.8  65.0  61.0 |  | 29.4/30.7 | 26.9  26.8  26.7 |  | Type 2 diabetes subjects | Age, sex, regional matched normal glucose tolerant controls |  | 1151 | 953 |
| Cauchi | 2006 | French | Caucasian | 1020 | 1402 | 57.1 | 58.6 |  | 30.0 | 22.8 |  | Type 2 diabetes subjects | Normoglycemic middle-aged nonobese controls |  | 2367 | 2499 |
| Groves | 2006 | UK | Caucasian | 1128 | 1291 | 64.1/60.2/46.3 | NR |  | 28.4/31.5/32.3 | NR |  | Patients with type 2 diabetes and excluded other type diabetes | British Birth Cohort of 1958 and Human Random Control |  | 2158 | 2574 |
| Hayashi | 2007 | Japan | East Asian | 978 | 638 | 61.5 | 45.5 |  | 23.7 | 22.9 |  | Type 2 diabetes patients recruited from the outpatient clinic | Nondiabetic controls were enrolled from an annual health check |  | 1630 | 1064 |
| Horikoshi | 2007 | Japan | East Asian | 123 | 129 | 62.8 | 68.6 |  | 23.9 | 24.0 |  | Type 2 diabetes patients randomly recruited from outpatient clinic in Tokyo, Japan | Nondiabetic controls from Health Management Center in Hiroshima, Japan |  | 192 | 272 |
| Horikoshi | 2007 | Japan | East Asian | 421 | 141 | 63.2 | 70.5 |  | 24.4 | 23.6 |  | Type 2 diabetes patients randomly recruited from outpatient clinic in Tokyo, Japan | Nondiabetic controls from Health Management Center in Hiroshima, Japan |  | 657 | 360 |
| Horikoshi | 2007 | Japan | East Asian | 139 | 99 | 70.6 | 68.6 |  | 24.0 | 23.8 |  | Type 2 diabetes patients randomly recruited from outpatient clinic in Hiroshima, Japan | Nondiabetic controls from Health Management Center in Hiroshima, Japan |  | 356 | 192 |
| Kimber | 2007 | UK | Caucasian | 1821 | 1720 | 60.7 | 65.3 |  | 31.3 | 26.8 |  | Type 2 diabetic patients from Go-DARTS2 study | Nondiabetic controls from Go-DARTS2 study |  | 3225 | 3291 |
| Marzi | 2007 | Germany | Caucasian | 393 | 870 | 62.5 | 60.8 |  | 30.3 | 28.1 |  | Type 2 diabetes subjects | Nondiabetic controls |  | 678 | 1691 |
| Mayans | 2007 | Sweden | North European | NR | NR | NR | NR |  | NR | NR |  | Type 2 diabetes subjects by clinical diagnosis | Age, sex and geographical matched controls without type 2 diabetes |  | 872 | 857 |
| van V-O * | 2007 | Dutch | Caucasian | 229 | 557 | 70.8 | 47.8 |  | 27.4 | NR |  | Type 2 diabetes patients diagnosed according to the WHO criteria | Healthy blood bank donors of white Dutch origin |  | 502 | 920 |
| Humphries | 2006 | UK | Caucasian | NR | NR | 64.9 | 56.0 |  | 29.4 | 26.1 |  | Type 2 diabetes patients diagnosed according to the WHO criteria | Healthy white men registered with nine primary care practices in UK |  | 1459 | 2518 |
| Humphries | 2006 | UK | Indian Asian | NR | NR | 58.7 | NR |  | 28.4 | 26.1 |  | Type 2 diabetes patients diagnosed according to the WHO criteria | Nondiabetic ethically matched controls from south London |  | 919 | 312 |
| Humphries | 2006 | UK | African | NR | NR | 61.6 | NR |  | 27.4 | 26.1 |  | Type 2 diabetes patients diagnosed according to the WHO criteria | Nondiabetic ethically matched controls from south London |  | 385 | 331 |
| Parra | 2007 | Mexican | Mexican American | 88 | 189 | 55.5 | 69.2 |  | 29.7 | 27.6 |  | Patients with T2D were recruited from their Primary Care in Mexican City | Normal glucose tolerant controls with no family history of diabetes |  | 375 | 275 |
| Bodhini | 2007 | India | Indian Asian | 426 | 474 | 41.0 | 49.0 |  | 25.1 | 23.6 |  | Type 2 diabetes subjects | Normal glucose tolerant controls |  | 1031 | 1038 |
| Chandak | 2007 | India | Indian Asian | 513 | 186 | 47.2 | 30.9 |  | 25.4 | 20.5 |  | Type 2 diabetes mellitus patients | Ethnical-matched controls with normal glucose tolerance |  | 955 | 399 |
| Chang | 2007 | China | East Asian | 429 | 282 | 60.0 | 64.5 |  | 24.7 | 23.6 |  | Type 2 diabetes subjects recruited from metabolism clinics in Taiwan | Normal glucose tolerant controls in routine health examination in Taiwan |  | 760 | 760 |
| Scott | 2007 | USA | North European | 653 | 574 | 63.4 | 64.0 |  | 29.8 | 26.8 |  | Type 2 diabetic patients in FUSHION 1 | Nondiabetic controls |  | 1161 | 1174 |
| Scott | 2007 | USA | North European | 724 | 768 | 60.0 | 59.0 |  | 30.1 | 26.4 |  | Type 2 diabetic patients in FUSHION 2 | Nondiabetic controls |  | 1215 | 1258 |
| Sladek | 2007 | France | Caucasian | 422 | 269 | 60.2 | 53.4 |  | 25.8 | 23.2 |  | Nonobese T2DM cases diagnosed with BMI<30 kg/m2 | NGT controls with BMI<27 kg/m2 |  | 694 | 654 |
| Sladek | 2007 | France | Caucasian | 1628 | 1240 | 62.2 | 56.4 |  | 28.9 | 25.3 |  | T2DM cases diagnosed with BMI<35 kg/m^2^ | NGT controls with BMI<35kg/m^2^ |  | 2617 | 2894 |
| Cauchi | 2007 | North African | African | 156 | 121 | 58.0 | 55.0 |  | 28.0 | 27.2 |  | Type 2 diabetes subjects | Normoglycemic individuals |  | 504 | 406 |
| Cauchi | 2007 | Austria | Caucasian | 285 | 725 | 56.5 | 51.5 |  | 30.7 | 26.4 |  | Type 2 diabetes subjects | Normoglycemic individuals |  | 486 | 1075 |
| da Silva † | 2007 | UK | Caucasian | 268 | 1061 | 67.4 | 63.9  /31.6 |  | 29.7 | 27.5  25.5 |  | Unrelated patients with type 2 diabetes and excluded type 1 diabetes | Normal glucose tolerant controls |  | 487 | 2099 |
| Miyake | 2007 | Japan | East Asian | 277 | 148 | 60.5 | 75.6 |  | 24.3 | 21.4 |  | Type 2 diabetic patients recruited from Hyogo, Japan | Nondiabetic controls |  | 465 | 323 |
| Miyake | 2007 | Japan | East Asian | 323 | 233 | 60.2 | 67.3 |  | 23.9 | 23.0 |  | Type 2 diabetic patients recruited from Gunma, Japan | Nondiabetic controls |  | 576 | 576 |
| Miyake | 2007 | Japan | East Asian | 664 | 420 | 62.5 | 69.2 |  | 23.1 | 22.6 |  | Type 2 diabetic patients recruited from seven districts  In Japan | Nondiabetic controls with no family history of diabetes in first- or second-degree relatives |  | 1173 | 974 |
| Rees | 2008 | Indian | Indian Asian | 450 | 219 | 56.9 | 55.0 |  | 28.3 | 28.1 |  | Type 2 diabetes subjects | Ethnically-matched Normoglycemic controls |  | 831 | 437 |
| Total |  |  |  |  |  |  |  |  |  |  |  |  |  |  | 35843 | 39123 |

¶ Excluded the subjects in family-based study.

* Abbreviation for van Vliet-Ostaptchouk

† Just community cases were selected; enriched cases repeated with another reference [60].

‡ NR, not reported.

**Table 2. Distribution of genotype of the rs7903146 polymorphism in studies of *TCF7L2*** gene and susceptibility to T2DM

| First author | Year | Ethnicity | Genotype distribution | | | | | | | | | | |  | Frequency of the risk allele (%) | |
| --- | --- | --- | --- | --- | --- | --- | --- | --- | --- | --- | --- | --- | --- | --- | --- | --- |
|  |  |  | cases | | | | |  | controls | | | | |  |  |  |
|  |  |  | N | TT | CT | CC | *P* value for HWE |  | N | TT | CT | CC | *P* value for HWE |  | Cases¶ | controls§ |
| Grant | 2006 | North European | 1066 | 173 | 490 | 403 | 0.505 |  | 788 | 87 | 302 | 399 | 0.383 |  | 39.2 | 30.2 |
| Grant | 2006 | North European | 214 | 29 | 93 | 92 | 0.779 |  | 498 | 43 | 187 | 268 | 0.453 |  | 35.3 | 27.4 |
| Grant | 2006 | Caucasian | 350 | 64 | 155 | 131 | 0.321 |  | 494 | 39 | 201 | 254 | 0.996 |  | 40.4 | 28.2 |
| Sale | 2007 | African | 577 | 63 | 294 | 220 | 0.0541 |  | 596 | 57 | 214 | 325 | 0.0512 |  | 36.4 | 27.6 |
| Guo | 2007 | American Pima Indian | 1304 | 11 | 169 | 1124 | 0.263 |  | 1657 | 16 | 226 | 1415 | 0.125 |  | 7.3 | 7.8 |
| Ng | 2007 | East Asian | 433 | 1 | 24 | 408 | 0.603 |  | 419 | 0 | 20 | 399 | 0.835 |  | 3.0 | 2.4 |
| Scott | 2006 | North European | 1113 | 58 | 381 | 674 | 0.910 |  | 920 | 26 | 277 | 617 | 0.746 |  | 22.3 | 17.9 |
| Cauchi | 2006 | Caucasian | 2367 | 431 | 1149 | 787 | 0.949 |  | 2499 | 231 | 1060 | 1208 | 0.998 |  | 42.5 | 30.5 |
| Groves | 2006 | Caucasian | 2001 | 270 | 960 | 771 | 0.570 |  | 2476 | 217 | 1084 | 1175 | 0.334 |  | 37.5 | 30.7 |
| Hayashi | 2007 | East Asian | 1619 | 4 | 165 | 1450 | 0.954 |  | 1067 | 2 | 85 | 980 | 0.994 |  | 5.3 | 4.2 |
| Horikoshi | 2007 | East Asian | 189 | 2 | 22 | 165 | 0.455 |  | 272 | 0 | 21 | 251 | 0.803 |  | 6.9 | 3.9 |
| Horikoshi | 2007 | East Asian | 650 | 2 | 64 | 584 | 0.985 |  | 360 | 2 | 20 | 338 | 0.500 |  | 5.2 | 3.2 |
| Horikoshi | 2007 | East Asian | 335 | 0 | 33 | 302 | 0.638 |  | 281 | 0 | 10 | 181 | 0.933 |  | 4.9 | 1.8 |
| Kimber | 2007 | Caucasian | 3224 | 361 | 1459 | 1404 | 0.826 |  | 3291 | 248 | 1329 | 1714 | 0.909 |  | 33.8 | 27.7 |
| Marzi | 2007 | Caucasian | 651 | 73 | 296 | 282 | 0.939 |  | 1641 | 121 | 678 | 842 | 0.623 |  | 33.9 | 28.0 |
| Mayans | 2007 | North European | 824 | 54 | 318 | 452 | 0.982 |  | 820 | 35 | 253 | 532 | 0.780 |  | 25.8 | 19.7 |
| van V-O * | 2007 | Caucasian | 496 | 72 | 221 | 203 | 0.645 |  | 907 | 83 | 365 | 459 | 0.699 |  | 36.8 | 29.3 |
| Humphries | 2006 | Caucasian | 1459 | 193 | 665 | 601 | 0.914 |  | 2493 | 197 | 1001 | 1295 | 0.983 |  | 36.0 | 28.0 |
| Humphries | 2006 | Indian Asian | 837 | 96 | 375 | 366 | 1.000 |  | 300 | 26 | 111 | 163 | 0.530 |  | 33.9 | 27.2 |
| Humphries | 2006 | African | 307 | 30 | 136 | 141 | 0.931 |  | 311 | 26 | 124 | 161 | 0.954 |  | 31.9 | 28.3 |
| Parra | 2007 | Mexican American | 371 | 11 | 100 | 261 | 0.982 |  | 271 | 7 | 73 | 191 | 1.000 |  | 19.9 | 16.1 |
| Bodhini | 2007 | Indian Asian | 1031 | 114 | 455 | 462 | 0.992 |  | 1038 | 92 | 391 | 555 | 0.159 |  | 33.1 | 26.7 |
| Chandak | 2007 | Indian Asian | 955 | 141 | 423 | 391 | 0.318 |  | 399 | 34 | 160 | 205 | 0.940 |  | 36.9 | 29.2 |
| Chang | 2007 | East Asian | 760 | 1 | 35 | 724 | 0.703 |  | 760 | 0 | 44 | 716 | 0.713 |  | 2.3 | 2.9 |
| Scott | 2007 | North European | 1161 | 55 | 422 | 684 | 0.614 |  | 1174 | 32 | 356 | 786 | 0.543 |  | 22.9 | 17.9 |
| Scott | 2007 | North European | 1172 | 68 | 393 | 711 | 0.236 |  | 1226 | 33 | 383 | 810 | 0.302 |  | 22.6 | 18.3 |
| Sladek | 2007 | Caucasian | 694 | 149 | 348 | 197 | 0.980 |  | 654 | 65 | 254 | 335 | 0.266 |  | 46.5 | 29.4 |
| Sladek | 2007 | Caucasian | 2499 | 408 | 1215 | 876 | 0.652 |  | 2849 | 238 | 1194 | 1417 | 0.854 |  | 40.6 | 29.3 |
| Cauchi | 2007 | African | 516 | 99 | 277 | 140 | 0.188 |  | 415 | 54 | 185 | 176 | 0.887 |  | 46.0 | 35.3 |
| Cauchi | 2007 | Caucasian | 486 | 78 | 208 | 200 | 0.163 |  | 1075 | 88 | 432 | 555 | 0.954 |  | 37.4 | 28.3 |
| da Silva | 2007 | Caucasian | 487 | 70 | 208 | 209 | 0.303 |  | 2099 | 180 | 887 | 1032 | 0.862 |  | 35.7 | 29.7 |
| Miyake | 2007 | East Asian | 462 | 1 | 38 | 423 | 0.989 |  | 323 | 0 | 18 | 305 | 0.876 |  | 4.3 | 2.9 |
| Miyake | 2007 | East Asian | 539 | 1 | 63 | 475 | 0.766 |  | 554 | 0 | 42 | 512 | 0.651 |  | 6.0 | 3.8 |
| Miyake | 2007 | East Asian | 1150 | 3 | 127 | 1020 | 0.901 |  | 957 | 1 | 77 | 879 | 0.876 |  | 5.9 | 4.1 |
| Rees | 2008 | Indian Asian | 836 | 116 | 360 | 352 | 0.305 |  | 432 | 44 | 166 | 222 | 0.303 |  | 35.4 | 29.4 |
| Total |  |  | 33135 | | | | |  | 36316 | | | | |  |  |  |

* Abbreviation for van Vliet-Ostaptchouk

¶ Pooled allele frequency for cases (%):26.1, 95 percent, 21.0, 31.2

§ Pooled allele frequency for controls (%): 20.2, 95 percent, 16.3, 24.1

**Table 3. Distribution of genotype of the rs7901695 polymorphism in studies of *TCF7L2*** gene and susceptibility to T2DM

| First author  (Ref. No.) | Year | Ethnicity | Genotype distribution | | | | | | | | | | |  | Frequency of the risk allele (%) | |
| --- | --- | --- | --- | --- | --- | --- | --- | --- | --- | --- | --- | --- | --- | --- | --- | --- |
|  |  |  | cases | | | | |  | controls | | | | |  |  |  |
|  |  |  | N | CC | CT | TT | *P* value for HWE |  | N | CC | CT | TT | *P* value for HWE |  | Cases¶ | controls§ |
| Grant | 2006 | North European | 1141 | 172 | 536 | 433 | 0.959 |  | 816 | 85 | 320 | 411 | 0.163 |  | 38.6 | 30.0 |
| Grant | 2006 | North European | 218 | 31 | 89 | 98 | 0.349 |  | 503 | 42 | 196 | 265 | 0.797 |  | 34.6 | 27.8 |
| Grant | 2006 | Caucasian | 353 | 61 | 163 | 129 | 0.744 |  | 513 | 41 | 220 | 252 | 0.765 |  | 40.4 | 29.4 |
| Sale | 2007 | African | 577 | 156 | 289 | 132 | 0.997 |  | 595 | 127 | 287 | 181 | 0.801 |  | 52.1 | 45.5 |
| Guo | 2007 | American Pima Indian | 1310 | 13 | 199 | 1098 | 0.501 |  | 1624 | 13 | 232 | 1379 | 0.646 |  | 8.6 | 7.9 |
| Scott | 2006 | North European | 1117 | 69 | 418 | 630 | 1.000 |  | 935 | 35 | 332 | 568 | 0.282 |  | 24.9 | 21.5 |
| Hayashi | 2007 | East Asian | 1617 | 4 | 173 | 1440 | 0.820 |  | 1056 | 2 | 88 | 966 | 1.00 |  | 5.6 | 4.4 |
| Horikoshi | 2007 | East Asian | 189 | 2 | 22 | 165 | 0.455 |  | 271 | 0 | 21 | 251 | 0.803 |  | 7.4 | 3.9 |
| Mayans | 2007 | North European | 825 | 62 | 329 | 434 | 0.999 |  | 793 | 38 | 286 | 469 | 0.800 |  | 27.5 | 22.8 |
| Rees | 2008 | Indian Asian | 828 | 111 | 355 | 362 | 0.276 |  | 437 | 42 | 169 | 226 | 0.457 |  | 34.8 | 28.9 |
| Total |  |  | 8175 | | | | |  | 7543 | | | | |  |  |  |

¶ Pooled allele frequency for cases (%):27.5, 95 percent, 16.1, 38.8

§Pooled allele frequency for controls (%): 22.2, 95 percent, 12.7, 31.7

**Table 4. Distribution of genotype of the rs12255372 polymorphism in studies of *TCF7L2*** gene and susceptibility to T2DM

| First author  (Ref. No.) | Year | Ethnicity | Genotype distribution | | | | | | | | | | |  | Frequency of the risk allele (%) | |
| --- | --- | --- | --- | --- | --- | --- | --- | --- | --- | --- | --- | --- | --- | --- | --- | --- |
|  |  |  | cases | | | | |  | controls | | | | |  |  |  |
|  |  |  | N | TT | GT | GG | *P* value for HWE |  | N | TT | GT | GG | *P* value for HWE |  | Cases¶ | controls§ |
| Grant | 2006 | North European | 1086 | 154 | 476 | 456 | 0.258 |  | 795 | 71 | 316 | 408 | 0.684 |  | 36.1 | 28.8 |
| Grant | 2006 | North European | 221 | 29 | 88 | 104 | 0.331 |  | 520 | 43 | 185 | 292 | 0.216 |  | 33.0 | 26.1 |
| Grant | 2006 | Caucasian | 344 | 62 | 144 | 138 | 0.846 |  | 509 | 39 | 186 | 284 | 0.546 |  | 39.0 | 25.9 |
| Sale | 2007 | African | 577 | 53 | 262 | 262 | 0.553 |  | 596 | 50 | 235 | 311 | 0.839 |  | 31.9 | 28.1 |
| Guo | 2007 | American Pima Indian | 1425 | 0 | 18 | 1407 | 0.972 |  | 1773 | 0 | 26 | 1747 | 0.953 |  | 2.8 | 1.7 |
| Zhang | 2006 | Caucasian | 1573 | 178 | 705 | 690 | 0.995 |  | 1947 | 140 | 772 | 1035 | 0.971 |  | 33.7 | 27.0 |
| Scott | 2006 | North European | 1128 | 50 | 354 | 724 | 0.726 |  | 936 | 20 | 255 | 661 | 0.727 |  | 20.1 | 15.8 |
| Cauchi | 2006 | Caucasian | 2367 | 373 | 1131 | 858 | 1.000 |  | 2483 | 209 | 1031 | 1243 | 0.973 |  | 39.6 | 29.2 |
| Groves | 2006 | Caucasian | 2021 | 244 | 941 | 836 | 0.704 |  | 2491 | 214 | 1057 | 1220 | 0.782 |  | 35.4 | 29.8 |
| Hayashi | 2007 | East Asian | 1630 | 3 | 112 | 1515 | 0.828 |  | 1043 | 0 | 45 | 998 | 0.776 |  | 3.6 | 2.2 |
| Horikoshi | 2007 | East Asian | 191 | 0 | 16 | 175 | 0.833 |  | 271 | 0 | 17 | 254 | 0.868 |  | 4.2 | 3.1 |
| Horikoshi | 2007 | East Asian | 657 | 1 | 41 | 615 | 0.935 |  | 360 | 1 | 16 | 343 | 0.246 |  | 3.3 | 2.5 |
| Horikoshi | 2007 | East Asian | 347 | 1 | 16 | 330 | 0.265 |  | 192 | 0 | 7 | 185 | 0.967 |  | 3.0 | 1.8 |
| Kimber | 2007 | Caucasian | 3225 | 359 | 1432 | 1434 | 0.999 |  | 3291 | 233 | 1323 | 1735 | 0.672 |  | 33.3 | 27.2 |
| Marzi | 2007 | Caucasian | 667 | 74 | 287 | 306 | 0.863 |  | 1658 | 107 | 698 | 853 | 0.0764 |  | 32.6 | 27.5 |
| Mayans | 2007 | North European | 828 | 38 | 333 | 457 | 0.0651 |  | 786 | 28 | 236 | 522 | 0.979 |  | 24.7 | 18.6 |
| van V-O * | 2007 | Caucasian | 492 | 59 | 217 | 216 | 0.924 |  | 901 | 84 | 348 | 469 | 0.256 |  | 34.0 | 28.6 |
| Humphries | 2006 | Caucasian | 1468 | 170 | 662 | 636 | 0.994 |  | 2489 | 188 | 994 | 1307 | 0.999 |  | 34.1 | 27.5 |
| Humphries | 2006 | Indian Asian | 841 | 68 | 350 | 423 | 0.933 |  | 302 | 20 | 104 | 178 | 0.668 |  | 28.9 | 23.8 |
| Humphries | 2006 | African | 304 | 28 | 120 | 156 | 0.780 |  | 313 | 24 | 148 | 141 | 0.214 |  | 28.9 | 31.3 |
| Parra | 2007 | Mexican American | 369 | 11 | 66 | 292 | 0.201 |  | 268 | 4 | 54 | 210 | 0.970 |  | 15.5 | 11.6 |
| Bodhini | 2007 | Indian Asian | 1031 | 66 | 348 | 617 | 0.210 |  | 1038 | 46 | 305 | 687 | 0.272 |  | 23.3 | 19.1 |
| Chandak | 2007 | Indian Asian | 955 | 99 | 377 | 479 | 0.160 |  | 399 | 22 | 134 | 243 | 0.825 |  | 30.1 | 22.3 |
| Chang | 2007 | East Asian | 760 | 0 | 9 | 751 | 0.987 |  | 760 | 0 | 6 | 754 | 0.994 |  | 0.6 | 0.4 |
| Sladek | 2007 | Caucasian | 694 | 131 | 342 | 221 | 0.998 |  | 654 | 55 | 267 | 332 | 0.992 |  | 43.5 | 28.8 |
| Miyake | 2007 | East Asian | 465 | 1 | 28 | 436 | 0.745 |  | 323 | 0 | 11 | 312 | 0.953 |  | 3.2 | 1.7 |
| Miyake | 2007 | East Asian | 559 | 2 | 48 | 509 | 0.753 |  | 565 | 0 | 27 | 538 | 0.844 |  | 4.8 | 2.4 |
| Miyake | 2007 | East Asian | 1146 | 2 | 76 | 1068 | 0.869 |  | 949 | 1 | 42 | 906 | 0.781 |  | 3.4 | 2.3 |
| Rees | 2008 | Indian Asian | 817 | 89 | 346 | 382 | 0.726 |  | 435 | 41 | 153 | 241 | 0.0791 |  | 32.1 | 27.0 |
| Total |  |  | 28188 |  |  |  |  |  | 29047 |  |  |  |  |  |  |  |

* Abbreviation for van Vliet-Ostaptchouk

¶ Pooled allele frequency for cases (%): 22.7, 95 percent: 17.2, 28.2

§ Pooled allele frequency for controls (%): 18.0, 95 percent: 13.5, 22.5

**Table 5. Distribution of genotype of the rs11196205 polymorphism in studies of *TCF7L2*** gene and susceptibility to T2DM

| First author  (Ref. No.) | Year | Ethnicity | Genotype distribution | | | | | | | | | | |  | Frequency of the risk allele (%) | |
| --- | --- | --- | --- | --- | --- | --- | --- | --- | --- | --- | --- | --- | --- | --- | --- | --- |
|  |  |  | cases | | | | |  | controls | | | | |  |  |  |
|  |  |  | N | CC | CG | GG | *P* value for HWE |  | N | CC | CG | GG | *P* value for HWE |  | Cases¶ | controls§ |
| Grant | 2006 | North European | 1061 | 303 | 517 | 241 | 0.772 |  | 781 | 185 | 370 | 226 | 0.379 |  | 52.9 | 47.4 |
| Grant | 2006 | North European | 220 | 60 | 105 | 55 | 0.801 |  | 514 | 97 | 256 | 161 | 0.965 |  | 51.1 | 43.8 |
| Grant | 2006 | Caucasian | 344 | 100 | 180 | 64 | 0.560 |  | 502 | 114 | 249 | 139 | 0.992 |  | 55.2 | 47.5 |
| Guo | 2007 | American Pima Indian | 1458 | 15 | 231 | 1212 | 0.566 |  | 1810 | 17 | 279 | 1514 | 0.587 |  | 9.0 | 8.6 |
| Ng | 2007 | East Asian | 433 | 1 | 22 | 410 | 0.492 |  | 419 | 0 | 11 | 408 | 0.964 |  | 2.8 | 1.3 |
| Scott | 2006 | North European | 1129 | 226 | 573 | 330 | 0.727 |  | 939 | 166 | 471 | 302 | 0.751 |  | 45.4 | 42.8 |
| Hayashi | 2007 | East Asian | 1595 | 13 | 198 | 1384 | 0.144 |  | 1061 | 2 | 107 | 952 | 0.855 |  | 7.0 | 5.2 |
| Horikoshi | 2007 | East Asian | 192 | 3 | 28 | 161 | 0.409 |  | 271 | 0 | 27 | 244 | 0.689 |  | 8.9 | 5.0 |
| Horikoshi | 2007 | East Asian | 657 | 3 | 76 | 578 | 0.958 |  | 360 | 3 | 38 | 319 | 0.315 |  | 6.2 | 6.1 |
| Horikoshi | 2007 | East Asian | 348 | 3 | 35 | 310 | 0.223 |  | 190 | 0 | 18 | 172 | 0.791 |  | 5.9 | 4.7 |
| Mayans | 2007 | North European | 818 | 131 | 412 | 275 | 0.527 |  | 787 | 117 | 388 | 282 | 0.679 |  | 41.2 | 39.5 |
| Miyake | 2007 | East Asian | 465 | 2 | 55 | 408 | 0.995 |  | 322 | 0 | 30 | 292 | 0.681 |  | 6.3 | 4.7 |
| Miyake | 2007 | East Asian | 539 | 7 | 77 | 455 | 0.325 |  | 544 | 1 | 58 | 485 | 0.865 |  | 8.0 | 5.5 |
| Miyake | 2007 | East Asian | 1170 | 6 | 153 | 1011 | 0.997 |  | 965 | 3 | 99 | 863 | 0.996 |  | 7.1 | 5.3 |
| Rees | 2008 | Indian Asian | 831 | 185 | 417 | 229 | 0.983 |  | 436 | 78 | 199 | 159 | 0.523 |  | 47.4 | 40.7 |
| Total |  |  | 11260 | | | |  |  | 9901 | | | |  |  |  |  |

¶ Pooled allele frequency for cases (%): 23.6, 95 percent: 11.7, 35.6

§ Pooled allele frequency for controls (%): 20.5, 95 percent: 9.7, 31.4

**Table 6. Summary odds ratios in the meta-analysis of contrasts of four SNPs polymorphisms in the *TCF7L2* gene and susceptibility to T2DM, by ethnic**

| Comparison | No. of studies | | Total sample size (n)* | Fixed effects | |  | Random effects | |  | Heterogeneity | | | |  | | Bayesian random effects | |
| --- | --- | --- | --- | --- | --- | --- | --- | --- | --- | --- | --- | --- | --- | --- | --- | --- | --- |
|  |  |  |  | Odds ratio | 95% confidence interval |  | Odds ratio | 95% confidence interval |  | *Q* | *P* value | *I*^2^ (%) | |  |  | Odds ratio | 95% credible interval |
| **rs7903146** | | | | | | | | | | | | | | | | | |
| **T allele vs. C allele** | **35** | | **33135/36316** | **1.439** | **1.403~1.477** |  | **1.416** | **1.346~1.491** |  | **104.966** | **0.000** | **67.6** | |  | | **1.418** | **1.354~1.483** |
| Caucasians | 11 | | 14714/20478 | 1.493 | 1.446~1.542 |  | 1.508 | 1.393~1.633 |  | 53.968 | 0.000 | 81.5 | |  | | 1.533 | 1.406~1.675 |
| French | 3 | | 5560/6002 | 1.713 | 1.622~1.809 |  | 1.762 | 1.578~1.968 |  | 7.096 | 0.029 | 71.5 | |  | | 1.766 | 1.637~1.943 |
| Other Caucasians | 8 | | 9154/14476 | 1.384 | 1.329~1.441 |  | 1.390 | 1.326~1.457 |  | 8.726 | 0.273 | 19.8 | |  | | 1.428 | 1.330~1.557 |
| North Europeans | 6 | | 5550/5426 | 1.383 | 1.298~1.474 |  | 1.383 | 1.298~1.474 |  | 2.473 | 0.781 | 0.0 | |  | | 1.263 | 1.045~1.632 |
| East Asians | 9 | | 6137/4993 | 1.408 | 1.228~1.614 |  | 1.430 | 1.204~1.698 |  | 11.300 | 0.185 | 29.2 | |  | | 1.444 | 1.225~1.741 |
| Indians | 4 | | 2169/3659 | 1.347 | 1.239~1.464 |  | 1.347 | 1.239~1.464 |  | 1.272 | 0.736 | 0.0 | |  | | 1.352 | 1.204~1.531 |
| Other racial groups | 5 | | 3075/3220 | 1.270 | 1.157~1.394 |  | 1.231 | 0.995~1.524 |  | 20.005 | 0.000 | 80.0 | |  | | 1.232 | 0.931~1.617 |
| Africans | 3 | | 1400/1322 | 1.451 | 1.295~1.625 |  | 1.436 | 1.237~1.667 |  | 3.373 | 0.185 | 40.7 | |  | | 1.432 | 1.224~1.715 |
| **TT vs. CC** | **35** | | **33135/36316** | **2.118** | **1.994~2.251** |  | **2.039** | **1.846~2.251** |  | **66.102** | **0.001** | **48.6** | |  | | **1.968** | **1.790~2.157** |
| Caucasians | 11 | | 14714/20478 | 2.283 | 2.123~2.456 |  | 2.313 | 1.992~2.684 |  | 37.578 | 0.000 | 73.4 | |  | | 2.303 | 1.961~2.689 |
| French | 3 | | 5560/6002 | 2.934 | 2.601~3.310 |  | 2.981 | 2.547~3.490 |  | 3.118 | 0.210 | 35.9 | |  | | 3.051 | 2.039~4.906 |
| Other Caucasians | 8 | | 9154/14476 | 1.974 | 1.800~2.163 |  | 1.988 | 1.796~2.199 |  | 8.093 | 0.324 | 13.5 | |  | | 1.989 | 1.807~2.216 |
| North Europeans | 6 | | 5550/5426 | 2.009 | 1.698~2.378 |  | 2.009 | 1.698~2.378 |  | 0.744 | 0.980 | 0.0 | |  | | 1.944 | 1.639~2.284 |
| East Asians | 9 | | 6137/4993 | 1.157 | 0.528~2.537 |  | 1.157 | 0.528~2.537 |  | 1.969 | 0.982 | 0.0 | |  | | 1.595 | 0.537~2.653 |
| Indians | 4 | | 2169/3659 | 1.682 | 1.393~2.030 |  | 1.682 | 1.393~2.030 |  | 2.144 | 0.543 | 0.0 | |  | | 1.669 | 1.373~2.104 |
| Other racial groups | 5 | | 3075/3220 | 1.639 | 1.298~2.069 |  | 1.545 | 1.124~2.123 |  | 6.512 | 0.164 | 35.6 | |  | | / | / |
| Africans | 3 | | 1400/1322 | 1.798 | 1.397~2.314 |  | 1.775 | 1.307~2.412 |  | 2.850 | 0.240 | 29.8 | |  | | 1.802 | 1.292~2.480 |
| **TC vs. CC** | **35** | | **33135/36316** | **1.422** | **1.373~1.473** |  | **1.414** | **1.330~1.503** |  | **84.028** | **0.000** | **59.5** | |  | | **1.406** | **1.341~1.476** |
| Caucasians | 11 | | 14714/20478 | 1.463 | 1.396~1.532 |  | 1.464 | 1.336~1.603 |  | 34.224 | 0.000 | 70.8 | |  | | 1.483 | 1.375~1.611 |
| French | 3 | | 5560/6002 | 1.718 | 1.587~1.860 |  | 1.793 | 1.526~2.107 |  | 7.025 | 0.030 | 71.5 | |  | | 1.766 | 1.437~2.278 |
| Other Caucasians | 8 | | 9154/14476 | 1.347 | 1.272~1.425 |  | 1.347 | 1.272~1.425 |  | 3.326 | 0.853 | 0.0 | |  | | 1.349 | 1.278~1.431 |
| North Europeans | 6 | | 5550/5426 | 1.356 | 1.250~1.471 |  | 1.362 | 1.235~1.502 |  | 7.013 | 0.220 | 28.7 | |  | | 1.357 | 1.255~1.474 |
| East Asians | 9 | | 6137/4993 | 1.377 | 1.194~1.588 |  | 1.381 | 1.178~1.620 |  | 9.328 | 0.315 | 14.2 | |  | | 1.352 | 1.160~1.591 |
| Indians | 4 | | 2169/3659 | 1.406 | 1.253~1.578 |  | 1.406 | 1.253~1.578 |  | 0.287 | 0.962 | 0.0 | |  | | 1.406 | 1.230~1.617 |
| Other racial groups | 5 | | 3075/3220 | 1.346 | 1.192~1.521 |  | 1.356 | 0.966~1.903 |  | 29.433 | 0.000 | 86.4 | |  | | / | / |
| Africans | 3 | | 1400/1322 | 1.764 | 1.498~2.076 |  | 1.714 | 1.301~2.259 |  | 5.531 | 0.063 | 63.8 | |  | | 1.696 | 1.258~2.242 |
| **rs12255372** | | | | | | | | | | | | | | | | | |
| **T allele vs. G allele** | **29** | | **28118/29047** | **1.383** | **1.343~1.425** |  | **1.379** | **1.307~1.454** |  | **66.191** | **0.000** | **57.7** | |  | | **1.381** | **1.311~1.452** |
| Caucasians | 9 | | 12851/16423 | 1.410 | 1.361~1.461 |  | 1.437 | 1.326~1.558 |  | 37.178 | 0.000 | 78.5 | |  | | 1.442 | 1.317~1.589 |
| Other Caucasians | 7 | | 9790/13286 | 1.345 | 1.292~1.400 |  | 1.352 | 1.279~1.430 |  | 10.258 | 0.114 | 41.5 | |  | | 1.418 | 1.286~1.589 |
| North Europeans | 4 | | 3263/3037 | 1.393 | 1.281~1.515 |  | 1.393 | 1.281~1.515 |  | 0.304 | 0.959 | 0.0 | |  | | 1.394 | 1.287~1.558 |
| East Asians | 8 | | 5755/4463 | 1.620 | 1.347~1.947 |  | 1.620 | 1.347~1.947 |  | 2.032 | 0.958 | 0.0 | |  | | 1.621 | 1.341~1.951 |
| Indians | 4 | | 3644/2174 | 1.328 | 1.214~1.453 |  | 1.328 | 1.214~1.453 |  | 1.963 | 0.580 | 0.0 | |  | | 1.332 | 1.162~1.535 |
| Other racial groups | 4 | | 2675/2950 | 1.065 | 0.936~1.211 |  | 1.042 | 0.881~1.231 |  | 4.192 | 0.241 | 28.4 | |  | | 1.030 | 0.748~1.342 |
| **TT vs. GG** | **29** | | **28118/29047** | **1.924** | **1.794~2.065** |  | **1.884** | **1.691~2.099** |  | **46.515** | **0.015** | **39.8** | |  | | **1.885** | **1.698~2.088** |
| Caucasians | 9 | | 12851/16423 | 2.039 | 1.878~2.213 |  | 2.096 | 1.784~2.462 |  | 27.921 | 0.000 | 71.3 | |  | | 2.091 | 1.755~2.528 |
| Other Caucasians | 7 | | 9790/13286 | 1.852 | 1.686~2.034 |  | 1.865 | 1.662~2.094 |  | 8.407 | 0.210 | 28.6 | |  | | 1.860 | 1.672~2.102 |
| North Europeans | 4 | | 3263/3037 | 1.905 | 1.536~2.364 |  | 1.905 | 1.536~2.364 |  | 1.105 | 0.776 | 0.0 | |  | | 1.889 | 1.485~2.412 |
| East Asians | 8 | | 5755/4463 | 1.191 | 0.476~2.980 |  | 1.191 | 0.476~2.980 |  | 1.228 | 0.990 | 0.0 | |  | | 1.875 | 0.464~3.439 |
| Indians | 4 | | 3644/2174 | 1.610 | 1.291~2.009 |  | 1.610 | 1.291~2.009 |  | 2.785 | 0.426 | 0.0 | |  | | 1.593 | 1.286~2.086 |
| Other racial groups | 4 | | 2675/2950 | 1.236 | 0.892~1.712 |  | 1.236 | 0.892~1.712 |  | 0.917 | 0.821 | 0.0 | |  | | / | / |
| **TG vs. GG** | **29** | | **28118/29047** | **1.369** | **1.315~1.425** |  | **1.367** | **1.288~1.450** |  | **47.980** | **0.011** | **41.6** | |  | | **1.360** | **1.291~1.433** |
| Caucasians | 9 | | 12851/16423 | 1.387 | 1.320~1.457 |  | 1.401 | 1.291~1.521 |  | 19.489 | 0.012 | 56.0 | |  | | 1.413 | 1.299~1.555 |
| Other Caucasians | 7 | | 9790/13286 | 1.323 | 1.251~1.399 |  | 1.323 | 1.251~1.399 |  | 4.327 | 0.633 | 0.0 | |  | | 1.326 | 1.251~1.412 |
| North Europeans | 4 | | 3263/3037 | 1.386 | 1.243~1.545 |  | 1.386 | 1.243~1.545 |  | 2.961 | 0.398 | 0.0 | |  | | 1.380 | 1.222~1.569 |
| East Asians | 8 | | 5755/4463 | 1.594 | 1.318~1.927 |  | 1.594 | 1.318~1.927 |  | 1.181 | 0.991 | 0.0 | |  | | 1.569 | 1.304~1.887 |
| Indians | 4 | | 3644/2174 | 1.362 | 1.212~1.532 |  | 1.362 | 1.212~1.532 |  | 0.867 | 0.833 | 0.0 | |  | | 1.358 | 1.176~1.579 |
| Other racial groups | 4 | | 2675/2950 | 1.022 | 0.864~1.210 |  | 0.949 | 0.689~1.309 |  | 9.143 | 0.027 | 56.3 | |  | | / | / |
| **rs7901695** | | | | | | | | | | | | | | | | | |
| C allele vs. T allele | 10 | | 8175/7543 | 1.322 | 1.248~1.400 |  | 1.323 | 1.232~1.419 |  | 12.615 | 0.181 | 28.7 | |  | | 1.324 | 1.236~1.422 |
| CC vs. TT | 10 | | 8175/7543 | 1.856 | 1.610~2.139 |  | 1.856 | 1.610~2.139 |  | 6.029 | 0.737 | 0.0 | |  | | 1.771 | 1.525~2.062 |
| CT vs. TT | 10 | | 8175/7543 | 1.286 | 1.191~1.389 |  | 1.290 | 1.184~1.405 |  | 10.816 | 0.289 | 16.8 | |  | | 1.292 | 1.199~1.402 |
| **rs11196205** | | | | | | | | | | | | | | | | | |
| C allele vs. G allele | | 15 | 11260/9901 | 1.215 | 1.152~1.218 |  | 1.238 | 1.153~1.330 |  | 20.968 | 0.102 | 33.2 |  | | 1.245 | | 1.166~1.341 |
| CC vs. GG | | 15 | 11260/9901 | 1.453 | 1.285~1.642 |  | 1.461 | 1.280~1.668 |  | 14.829 | 0.390 | 5.6 |  | | 1.471 | | 1.300~1.706 |
| CG vs. GG | | 15 | 11260/9901 | 1.223 | 1.135~1.318 |  | 1.223 | 1.135~1.318 |  | 12.874 | 0.536 | 0.0 |  | | 1.242 | | 1.152~1.355 |

* Cases/control
